# Supplementary material for: Vasohibin1, a new mouse cardiomyocyte IRES trans-acting factor that regulates translation in early hypoxia
Source: eLife. 2019 Dec 9;8:e50094. doi: 10.7554/eLife.50094 (PMC6946400; doi:10.7554/eLife.50094)
Supplement: Supplementary file 1. — Total RNA was purified from HL-1 cardiomyocytes submitted to increasing periods from 5 min to 24 hr of hypoxia at 1% O2, as well as from normoxic cardiomyocytes as a control. cDNA was synthesized and used for a Fluidigm deltagene PCR array dedicated to genes related to (lymph)angiogenesis or stress (Supplementary file 6). Analysis was performed in three biological replicates (cell culture well and cDNA), each of them measured in three technical replicates (PCR reactions). Relative quantification (RQ) of gene expression in hypoxia was calculated using the 2–ΔΔCT method with normalization to 18S and to normoxia. Standard deviation is indicated. When the RQ value is inferior to 1, the fold change is expressed as −1/RQ. ND means ‘non detected’. '–' means that the gene was not included in the array. [file elife-50094-supp1.docx]

Hantelys et al, Supplementary File 1

| **Hypoxia time** | **5min** |  | **30min** |  | **1h** |  | **2h** |  | **4h** |  | **8h** |  | **24h** |  |
| --- | --- | --- | --- | --- | --- | --- | --- | --- | --- | --- | --- | --- | --- | --- |
| **Gene name** | **RQ** |  | **RQ** |  | **RQ** |  | **RQ** |  | **RQ** |  | **RQ** |  | **RQ** |  |
| *Akt1* | 1.60 | ±0.13 | 1.30 | ±0.06 | 1.20 | ±0.15 | 1.36 | ±0.25 | -1.10 | ±0.09 | 1.40 | ±0.13 | 1.00 | ±0.32 |
| *Ang* | 2.65 | ±0.25 | 1.23 | ±0.36 | -1.04 | ±0.12 | 1.24 | ±0.04 | 1.24 | ±0.12 | 1.09 | ±0.08 | 1.26 | ±0.28 |
| *Angpt1* | 5.51 | ±0.17 | 1.20 | ±0.30 | 1.19 | ±0.07 | 1.90 | ±0.49 | 2.43 | ±0.36 | 1.56 | ±0.43 | -2.27 | ±0.06 |
| *Angptl4* | 1.03 | ±0.02 | -3.14 | ±0.09 | 1.65 | ±0.54 | 1.60 | ±0.46 | 1.56 | ±0.67 | 3.50 | ±1.07 | -1.37 | ±0.35 |
| *Anpep* | ND |  | -3.26 | ±0.29 | 1.65 | ±0.01 | -8.05 | ±0.06 | -1.04 | ±0.14 | 1.62 | ±0.62 | 1.03 | ±0.43 |
| *Apln (Apelin)* | 2.11 |  | -19,21 | ±0.01 | -6.17 | ±0.05 | -29.04 | ±0.01 | 36.77 | ±5.59 | 109.98 | ±6.98 | 335.72 | ±57.52 |
| *Aplnr* | ND |  | -2.36 | ±0.03 | -1.57 | ±0.10 | 1.44 | ±0.32 | 3.25 | ±1.70 | 1.98 | ±0.72 | 2.31 | ±1.29 |
| *Atp2a2 (SERCA2)* | 1.46 | ±0.08 | 1.21 | ±0.13 | 1.26 | ±0.14 | 1.50 | ±0.01 | 1.20 | ±0.06 | 1.84 | ±0.18 | 1.48 | ±0.32 |
| *Bai1* | -1.52 | ±0.19 | 2.90 | ±0.91 | 1.17 | ±0.06 | 1.65 | ±0.52 | 1.36 | ±0.13 | 2.05 | ±0.60 | 1.04 | ±0.30 |
| *Ccl2* | ND |  | -1.09 | ±0.49 | 1.76 | ±0.46 | -2.76 | ±0.16 | 1.57 | ±1.01 | 2.99 | ±1.13 | 1.23 |  |
| *Ccl21a* | ND |  | -32.39 | ±0.01 | 2.97 | ±0.85 | -1.90 | ±0.03 | 2.65 | ±2.78 | -2.27 | ±0.21 | 1.32 | ±0.39 |
| *Col18a1* | 1.66 | ±0.71 | 1.58 | ±0.39 | 1.14 | ±0.14 | 1.45 | ±0.04 | -1.69 | ±0.09 | 1.82 | ±0.43 | 1.57 | ±0.43 |
| *Col4a3* | 19.64 | ±0.07 | 1.52 | ±0.19 | 1.20 | ±0.21 | 1.31 | ±0.04 | 1.00 | ±0.03 | 1.45 | ±0.30 | 4.40 | ±0.80 |
| *Ctgf* | 1.29 | ±0.09 | 1.67 | ±0.26 | 1.16 | ±0.06 | 1.30 | ±0.13 | 1.06 | ±0.13 | 2.05 | ±0.30 | 3.66 | ±0.76 |
| *Cxcl1* | 1.32 | ±0.64 | 1.11 | ±0.27 | 1.67 | ±0.19 | 1.58 | ±0.12 | 1.01 | ±0.09 | -1.43 | ±0.08 | -2.04 | ±0.13 |
| *Cxcl10* | 1.37 | ±0.86 | 1.36 | ±0.29 | 1.11 | ±0.14 | 1.33 | ±0.25 | 1.05 | ±0.09 | -1.02 | ±0.13 | -1.08 | ±0.23 |
| *Cyr61* | 2.30 | ±0.18 | 3.14 | ±0.14 | 1.59 | ±0.18 | -1.18 | ±0.07 | -1.03 | ±0.06 | 1.56 | ±0.12 | 1.15 | ±0.21 |
| *Edn1* | -2.43 | ±0.28 | 2.93 | ±0.31 | 1.72 | ±0.32 | -1.4 | ±0.24 | -1.39 | ±0.06 | -1.33 | ±0.09 | 1.04 | ±0.26 |
| *Efna1* | 2.08 | ±0.15 | 1.23 | ±0.10 | -1.52 | ±0.12 | -1.16 | ±0.19 | 1.25 | ±0.09 | 1.17 | ±0.05 | 1.35 | ±0.33 |
| *Efnb2* | 1.74 | ±0.14 | 1.55 | ±0.25 | 1.14 | ±0.23 | -1.03 | ±0.18 | 1.57 | ±0.18 | 3.71 | ±0.61 | 1.65 | ±0.42 |
| *Egf* | ND |  | 1.35 | ±0.14 | 1.32 | ±0.30 | 1.56 | ±0.44 | 1.56 | ±0.11 | 2.14 | ±0.53 | -1.61 | ±0.35 |
| *Eng* | 2.96 | ±0.94 | 1.55 | ±0.39 | 1.18 | ±0.19 | -1.02 | ±0.14 | -1.11 | ±0.15 | 2.27 | ±0.33 | 1.65 | ±0.34 |
| *Ephb4* | 2.22 | ±0.13 | 1.67 | ±0.16 | -1.03 | ±0.14 | 1.10 | ±0.13 | 1.01 | ±0.12 | 1.45 | ±0.09 | 1.57 | ±0.21 |
| *Erbb2 (Her2)* | 1.87 | ±0.19 | 1.85 | ±0.26 | -1.01 | ±0.18 | 1.16 | ±0.18 | 1.32 | ±0.11 | 1.00 | ±0.18 | 1.34 | ±0.09 |
| *F3* | 1.12 | ±0.94 | 1.45 | ±0.18 | 1.28 | ±0.11 | -1.57 | ±0.23 | -1.23 | ±0.07 | -1.09 | ±0.10 | -1.56 | ±0.21 |
| *Fgf1 (aFGF)* | -2.63 | ±0.18 | 1.69 | ±0.23 | 1.29 | ±0.22 | 1.37 | ±0.21 | -1.32 | ±0.05 | -1.43 | ±0.20 | 1.08 | ±0.20 |
| *Fgf2 (bFGF)* | 1.06 | ±0.04 | 1.76 | ±0.13 | -2.5 | ±0.03 | ND |  | -16.05 | ±0.02 | -2.05 | ±0.07 | 1.43 | ±0.35 |
| *Fgfr3* | 1.41 | ±0.07 | 1.34 | ±0.27 | -1.08 | ±0.12 | 1.38 | ±0.31 | 1.47 | ±0.27 | 3.93 | ±0.55 | 2.69 | ±0.39 |

| ***Hypoxia time*** | **5min** |  | **30min** |  | **1h** |  | **2h** |  | **4h** |  | **8h** |  | **24h** |  |
| --- | --- | --- | --- | --- | --- | --- | --- | --- | --- | --- | --- | --- | --- | --- |
| ***Gene name*** | **RQ** |  | **RQ** |  | **RQ** |  | **RQ** |  | **RQ** |  | **RQ** |  | **RQ** |  |
| *Fn1* | 3.30 | ±0.07 | 1.70 | ±0.15 | -1.02 | ±0.17 | 1.16 | ±0.01 | -1.25 | ±0.06 | 2.45 | ±0.36 | 2.61 | ±0.49 |
| *Hif1a* | 2.97 | ±0.03 | 1.20 | ±0.24 | 1.28 | ±0.18 | -1.42 | ±0.26 | -1.15 | ±0.08 | -1.06 | ±0.21 | -1.75 | ±0.22 |
| *Hnrnpm* | 1.55 | ±0.18 | 1.17 | ±0.18 | 1.54 | ±0.15 | 2.26 | ±0.20 | 1.86 | ±0.25 | 3.86 | ±1.33 | 1.23 | ±0.26 |
| *Hpse* | ND |  | 3.23 | ±1.07 | 1.25 | ±0.34 | 1.64 | ±0.16 | 1.56 | ±0.14 | 3.52 | ±1.07 | 11.04 | ±1.95 |
| *Id1* | -1.61 | ±1.12 | 1.58 | ±0.18 | 2.63 | ±0.50 | 3.01 | ±1.27 | 4.23 | ±0.19 | 2.01 | ±0.40 | 1.79 | ±0.64 |
| *Ifna1* | ND |  | 1.72 | ±0.17 | -1.09 | ±0.06 | 1.73 | ±0.07 | 2.19 | ±0.33 | 3.11 | ±0.25 | 1.31 | ±0.49 |
| *Igf1* | 10.07 | ±0.59 | 1.54 | ±0.22 | 1.42 | ±0.15 | 1.06 | ±0.04 | 1.10 | ±0.22 | -1.25 | ±0.14 | -2.86 | ±0.14 |
| *Igf1r* | - |  | - |  | - |  | - |  | -3.17 | ±0.08 | 2.37 | ±0.20 | 5.14 | ±0.32 |
| *Itgav* | ND |  | 1.19 | ±0.11 | -1.36 | ±0.14 | 1.33 | ±0.29 | 1.33 | ±0.12 | 3.41 | ±0.56 | 2.82 | ±0.64 |
| *Itgb3* | -4.33 | ±0.30 | 1.34 | ±0.19 | -1.35 | ±0.02 | 1.04 | ±0.20 | 1.03 | ±0.20 | 1.42 | ±0.34 | -1.37 | ±0.10 |
| *Jag1* | 2.13 | ±0.09 | 1.80 | ±0.24 | 1.03 | ±0.13 | 1.66 | ±0.46 | 1.46 | ±0.06 | 2.26 | ±0.37 | 1.76 | ±0.21 |
| *Mdk* | 1.14 | ±0.21 | 2.45 | ±0.17 | -1.07 | ±0.17 | -1.27 | ±0.00 | 1.16 | ±0.32 | 1.52 | ±0.38 | -1.16 | ±0.46 |
| *Mmp14* | -1.77 | ±0.03 | 1.30 | ±0.22 | 1.02 | ±0.06 | -1.16 | ±0.17 | 1.06 | ±0.11 | 1.70 | ±0.24 | 1.95 | ±0.15 |
| *Mmp2* | 1.46 | ±0.18 | 1.96 | ±0.50 | 1.20 | ±0.05 | 1.68 | ±0.44 | 1.14 | ±0.33 | 1.94 | ±0.66 | -1.23 | ±0.37 |
| *Neat1* | 4.03 | ±0.48 | -1.17 | ±0.23 | -1.59 | ±0.05 | 2.65 | ±0.00 | 1.89 | ±0.15 | 2.96 | ±1.02 | -1.16 | ±0.29 |
| *Nos3* | 9.29 |  | -4.01 | ±0.10 | 2.00 | ±0.54 | -2.58 | ±0.26 | -1.30 | ±0.14 | -1.02 | ±0.40 | -1.32 | ±0.19 |
| *Nrp1* | 2.46 | ±0.06 | 1.32 | ±0.17 | 1.04 | ±0.15 | -1.25 | ±0.25 | -1.10 | ±0.07 | 1.47 | ±0.25 | 1.10 | ±0.16 |
| *Nrp2* | 2.50 | ±0.07 | 1.55 | ±0.05 | 1.02 | ±0.12 | 1.02 | ±0.12 | 1.14 | ±0.19 | 1.72 | ±0.32 | 1.83 | ±0.39 |
| *P54nrb* | 2.96 | ±0.09 | 1.58 | ±0.42 | 1.21 | ±0.20 | 1.42 | ±0.29 | 1.22 | ±0.13 | 1.66 | ±0.30 | 1.11 | ±0.23 |
| *PAI1 (SerpinE1)* | -1.73 | ±0.12 | 2.18 | ±0.54 | 1.46 | ±0.15 | 1.74 | ±0.65 | 5.75 | ±0.57 | 15.76 | ±2.01 | 13.18 | ±1.02 |
| *Pdgfa* | 3.65 | ±0.43 | 1.45 | ±0.18 | 1.29 | ±0.26 | 1.59 | ±0.36 | 1.71 | ±0.11 | 1.97 | ±0.35 | 1.54 | ±0.34 |
| *Pecam1* | 1.48 |  | -11.55 | ±0.02 | -1.09 | ±0.24 | 2.86 | ±1.08 | 2.09 | ±0.77 | 2.70 | ±1.72 | 2.60 | ±0.86 |
| *Pf4* | ND |  | -39.36 | ±0.02 | -1.79 | ±0.33 | -2.20 | ±0.18 | 1.80 | ±0.40 | 1.48 | ±0.55 | 1.01 | ±0.28 |
| *Pgf* | ND |  | 2.17 | ±0.14 | -1.36 | ±0.19 | -7.89 | ±0.09 | 4.47 | ±0.89 | 1.85 | ±0.29 | 3.22 | ±0.85 |
| *Plau (upa)* | 1.13 | ±0.29 | -1.04 | ±0.24 | -1.36 | ±0.16 | -1.31 | ±0.13 | 1.25 | ±0.30 | 1.58 | ±0.26 | 1.24 | ±0.24 |
| *Plg* | ND |  | -2.71 | ±0.26 | -1.05 |  | 2.45 | ±0.63 | -1.35 | ±0.19 | -3.33 | ±0.16 | -2.94 | ±0.25 |
| *Prox1* | 7.13 | ±0.20 | 1.48 | ±0.23 | 1.26 | ±0.14 | -1.13 | ±0.14 | -1.08 | ±0.13 | 1.27 | ±0.29 | -1.72 | ±0.07 |

| ***Hypoxia time*** | **5min** |  | **30min** |  | **1h** |  | **2h** |  | **4h** |  | **8h** |  | **24h** |  |
| --- | --- | --- | --- | --- | --- | --- | --- | --- | --- | --- | --- | --- | --- | --- |
| ***Gene name*** | **RQ** |  | **RQ** |  | **RQ** |  | **RQ** |  | **RQ** |  | **RQ** |  | **RQ** |  |
| *Psf/Sfpq* | 1.45 | ±0.17 | 1.37 | ±0.09 | 1.30 | ±0.19 | 1.22 | ±0.08 | 1.10 | ±0.15 | 1.55 | ±0.59 | -1.43 | ±0.16 |
| *Pspc1* | 1.00 | ±0.80 | 1.34 | ±0.11 | 1.50 | ±0.01 | 1.64 | ±0.58 | 1.67 | ±0.19 | 1.37 | ±0.31 | 1.59 | ±0.23 |
| *SerpinF1* | ND |  | -31.89 | ±0.02 | -9.09 | ±0.01 | -1.91 | ±0.33 | -2.38 | ±0.11 | -1.14 | ±0.17 | 1.02 | ±0.37 |
| *Sphk1* | ND |  | 1.91 | ±0.80 | 1.70 | ±0.04 | -1.51 | ±0.19 | 1.30 | ±0.11 | 2.78 | ±1.20 | 1.17 | ±0.43 |
| *Tek* | 1.97 | ±1.55 | 1.98 | ±0.23 | 1.55 | ±0.41 | 1.48 | ±0.20 | 1.24 | ±0.14 | -1.25 | ±0.09 | 1.04 | ±0.13 |
| *Tgfa* | ND |  | -2.01 | ±0.13 | -7.94 | ±0.05 | -6.9 | ±0.03 | 2.91 | ±0.63 | 3.50 | ±1.29 | 4.05 | ±1.71 |
| *Tgfb1* | -5.26 | ±0.02 | 1.22 | ±0.23 | -1.45 | ±0.14 | 1.03 | ±0.10 | -1.02 | ±0.22 | 1.45 | ±0.24 | 1.05 | ±0.25 |
| *Tgfb2* | -2.45 | ±0.08 | 1.50 | ±0.31 | 1.22 | ±0.07 | 1.37 | ±0.12 | 1.78 | ±0.24 | 2.00 | ±0.39 | 1.43 | ±0.40 |
| *Tgfbr1* | -2.03 | ±0.57 | 1.33 | ±0.16 | 1.28 | ±0.25 | 1.48 | ±0.14 | 1.86 | ±0.34 | 2.23 | ±0.77 | -1.27 | ±0.16 |
| *Thbs1* | 2.65 | ±0.09 | 1.88 | ±0.12 | 1.19 | ±0.09 | 1.47 | ±0.33 | 1.12 | ±0.07 | 1.30 | ±0.22 | 1.44 | ±0.08 |
| *Thbs2* | 2.15 | ±0.12 | 1.52 | ±0.10 | 1.03 | ±0.16 | 1.20 | ±0.18 | -1.20 | ±0.01 | 1.21 | ±0.15 | -1.22 | ±0.21 |
| *Timp1* | -2.20 | ±0.04 | 1.13 | ±0.27 | -1.32 | ±0.29 | -1.96 | ±0.17 | -1.28 | ±0.12 | -1.22 | ±0.11 | -2.33 | ±0.19 |
| *Timp2* | 2.21 | ±0.04 | 1.46 | ±0.22 | 1.14 | ±0.01 | 1.13 | ±0.09 | 1.67 | ±0.13 | 1.28 | ±0.12 | -1.69 | ±0.15 |
| *Timp3* | ND |  | 2.70 | ±0.43 | 1.40 | ±0.23 | 1.98 | ±0.46 | 1.09 | ±0.16 | 1.04 | ±0.29 | 1.11 | ±0.17 |
| *Vash1* | - |  | - |  | - |  | - |  | -3.01 | ±0.14 | 1.56 | ±0.30 | 1.10 | ±0.08 |
| *Vegfa* | 2.90 | ±0.02 | 1.40 | ±0.18 | 1.20 | ±0.12 | 1.72 | ±0.50 | 4.48 | ±0.43 | 9.12 | ±1.48 | 4.71 | ±0.69 |
| *Vegfb* | 1.38 | ±0.04 | 1.33 | ±0.10 | -1.2 | ±0.11 | 1.02 | ±0.04 | -1.09 | ±0.07 | 1.04 | ±0.22 | -1.59 | ±0.10 |
| *Vegfc* | ND |  | ND |  | 1.38 | ±0.18 | ND |  | -5.03 | ±0.05 | -11.37 | ±0.03 | -2.86 | ±0.16 |
| *Vegfd* | ND |  | -1.19 | ±0.17 | -1.73 | ±0.22 | -1.99 | ±0.23 | 1.05 | ±0.37 | 1.48 | ±0.29 | 1.08 | ±0.24 |
| *Vegfr2 (Kdr)* | -2.05 | ±0.05 | 1.25 | ±0.13 | 1.07 | ±0.04 | 1.43 | ±0.21 | 1.31 | ±0.10 | 2.29 | ±0.38 | -1.01 | ±0.30 |
