# Supplementary material for: Vasohibin1, a new mouse cardiomyocyte IRES trans-acting factor that regulates translation in early hypoxia
Source: eLife. 2019 Dec 9;8:e50094. doi: 10.7554/eLife.50094 (PMC6946400; doi:10.7554/eLife.50094)
Supplement: Supplementary file 2. — Polysomes were purified on a sucrose gradient from HL-1 cardiomyocytes, either in normoxia or after 4 hr or 24 hr of hypoxia at 1% O2, as described in 'Materials and Methods'. RNA was purified from polysome-bound fractions and from cell lysate (before gradient loading). cDNA and PCR arrays were performed as in Figure 1 and in Supplementary file 1. Relative quantification (RQ) of gene expression in hypoxia was calculated using the 2-ΔΔCT method (polysomal RNA/total RNA normalized to normoxia). The 4 hr of hypoxia array was repeated in two independent arrays (RQ1 and RQ2). The values presented in Figures 2 and 3 correspond to RQ1 values. In Figure 6A and B, values are from RQ2. For RQ1, gene expression analysis was performed in three biological replicates (cell culture well and cDNA), each of them measured in three technical replicates (PCR reactions). For RQ2 (4 hr and 24 hr), analysis was performed in two biological replicates, each of them measured in two technical replicates. Standard deviation is indicated. When the RQ value is inferior to 1, the fold change is expressed as −1/RQ. ND means ‘non detected’. '–' means that the gene was not included in the array. [file elife-50094-supp2.docx]

Hantelys et al. Supplementary File 2

**Hypoxia: 4 h**

|  | **Total mRNA** | | **Polysome bound mRNA** | | **Fold change** | | **Standard deviation** | |
| --- | --- | --- | --- | --- | --- | --- | --- | --- |
| **Gene name** | RQ 1 | RQ 2 | RQ 1 | RQ 2 | RQ(polysomes)/  RQ(total mRNA) 1 | RQ(polysomes)/  RQ(total mRNA) 2 | ST DEV 1 | ST DEV 2 |
| *Akt1* | 0.54 | 0.63 | 1.12 | 1.45 | 2.07 | 2.29 | 0.07 | 0.05 |
| *Ang* | 0.22 | 0.59 | 0.54 | 0.44 | 2.42 | -1.35 | 0.53 | 0.01 |
| *Angpt1* | 0.16 | 0.48 | 0.37 | 0.32 | 2.35 | -1.48 | 0.67 | 0.01 |
| *Angptl4* | 0.21 | 0.39 | 0.67 | 0.48 | 3.26 | 1.23 | 1.56 | 0.15 |
| *Anpep* | 0.41 | 0.40 | 1.39 | 1.59 | 3.41 | 3.96 | 0.21 | 0.83 |
| *Apelin* | 35.38 | 2.85 | 60.39 | 4.47 | 1.71 | 1.57 | 0.27 | 0.10 |
| *Aplnnr* | 3.25 | ND | 1.12 | ND | -2.86 | ND | 0.35 | ND |
| *Atp2a2* | 0.31 | 0.45 | 0.6 | 0.84 | 1.94 | 1.84 | 0.34 | 0.07 |
| *Bai1* | 0.16 | 0.39 | 0.61 | 1.97 | 3.8 | 5.06 | 0.89 | 0.30 |
| *Ccl2* | 1.57 | 1.96 | ND | ND | ND | ND | ND | ND |
| *Ccl21a* | 2.65 | ND | ND | ND | ND | ND | ND | ND |
| *Col18a1* | 0.45 | 0.57 | 1.18 | 0.88 | 2.64 | 1.53 | 0.61 | 0.19 |
| *Col4a3* | 0.37 | 0.51 | 0.87 | 1.15 | 2.36 | 2.24 | 0.41 | 0.13 |
| *Ctgf* | 0.29 | 0.48 | 0.9 | 0.13 | 3.15 | -3.77 | 0.49 | 0.00 |
| *Cxcl1* | 0.1 | 0.56 | 0.22 | 0.29 | 2.21 | -1.95 | 0.3 | 0.04 |
| *Cxcl10 (inp10)* | 0.19 | 0.43 | 0.74 | 0.13 | 3.96 | -3.26 | 1.43 | 0.02 |
| *Cyr 61* | 0.45 | 0.72 | 0.91 | 0.62 | 2 | -1.16 | 0.24 | 0.04 |
| *Edn1* | 0.27 | 0.48 | 0.68 | 0.68 | 2.49 | 1.42 | 0.55 | 0.03 |
| *Efna1* | 0.3 | 0.48 | 0.69 | 0.82 | 2.29 | 1.69 | 0.29 | 0.16 |
| *Efnb2* | 0.53 | 0.55 | 1.31 | 0.68 | 2.48 | 1.23 | 0.71 | 0.01 |
| *Egf* | 0.21 | 0.56 | 0.53 | 2.34 | 2.46 | 4.16 | 1.56 | 0.67 |
| *Eng* | 0.8 | 0.63 | 2.16 | 1.36 | 2.71 | 2.16 | 0.78 | 0.25 |
| *Ephb4* | 0.31 | 0.50 | 0.58 | 0.68 | 1.87 | 1.36 | 0.23 | 0.05 |
| *Erbb2(her2)* | 0.27 | 0.41 | 0.55 | 0.60 | 2.02 | 1.44 | 0.24 | 0.08 |
| *F3* | 0.44 | 0.78 | 1.1 | 0.51 | 2.48 | -1.53 | 0.51 | 0.00 |
| *Fgf1* | 0.3 | 0.40 | 0.76 | 1.16 | 2.56 | 2.90 | 0.87 | 0.19 |
| *Fgf2* | 0.06 | - | ND | - | ND | - | ND | - |
| *Fgfr3* | 0.63 | 0.70 | 1.35 | 0.88 | 2.15 | 1.25 | 0.41 | 0.12 |
| *Fibrillarin* | ND | 0.61 | ND | 1.06 | ND | 1.73 | ND | 0.04 |
| *Fn1* | 0.48 | 0.46 | 1.13 | 0.57 | 2.34 | 1.23 | 0.3 | 0.04 |
| *Hif1a* | 0.52 | 0.55 | 0.97 | 0.90 | 1.88 | 1.63 | 0.36 | 0.18 |
| *Hif2a* | ND | 0.39 | ND | 0.64 | ND | 1.64 | ND | 0.14 |
| *Hnrnpm* | 0.74 | 0.68 | 1.84 | 1.42 | 2.48 | 2.08 | 0.5 | 0.01 |
| *Hpse* | 0.66 | 0.61 | 2.75 | 1.26 | 4.17 | 2.07 | 0.79 | 0.16 |
| *Id1* | 0.47 | ND | 0.9 | 0.87 | 1.93 | ND | 0.38 | ND |
| *Ifna1* | 0.34 | ND | 1.35 | 1.91 | 3.93 | ND | 1.38 | ND |
| *Igf1* | 0.22 | 0.40 | 0.44 | 0.79 | 2.03 | 1.97 | 0.54 | 0.13 |
| *Igf1r* | 1.05 | 0.79 | 1.97 | 1.49 | 1.88 | 1.88 | 0.06 | 0.06 |
| *Itgav* | 0.48 | 0.61 | 0.86 | 0.75 | 1.79 | 1.22 | 0.31 | 0.01 |
| *Itgb3* | 0.48 | 0.56 | 1.18 | 0.52 | 2.44 | -1.07 | 0.4 | 0.09 |
| *Jag1* | 0.17 | ND | 0.35 | ND | 2.03 | ND | 0.38 | ND |
| *Mdk* | 0.47 | ND | 0.96 | 0.66 | 2.03 | ND | 0.35 | ND |
| *Mmp14* | 0.44 | 0.79 | 0.82 | 0.90 | 1.85 | 1.13 | 0.18 | 0.13 |
| *Mmp2* | 0.27 | 0.24 | 0.52 | 1.26 | 1.91 | 5.14 | 0.44 | 2.48 |
| *Neat-1* | 0.22 | 0.43 | 0.88 | 4.21 | 3.98 | 9.90 | 1.24 | 0.03 |
| *Nos3* | 1.08 | 0.20 | 0.84 | 2.35 | -1.30 | 11.86 | 0.1 | 4.49 |
| *Nrp1* | 0.54 | 0.48 | 0.9 | 0.35 | 1.67 | -1.37 | 0.22 | 0.00 |
| *Nrp2* | 0.51 | 0.60 | 0.96 | 0.78 | 1.88 | 1.29 | 0.18 | 0.09 |
| *P54nrb* | 0.57 | 0.73 | 1.77 | 1.32 | 3.12 | 1.81 | 0.78 | 0.09 |
| *Pai-1* | 4.77 | 2.31 | 9.15 | 5.12 | 1.92 | 2.22 | 0.27 | 0.27 |
| *Pdgfa* | 0.41 | 0.59 | 0.99 | 1.33 | 2.42 | 2.24 | 0.48 | 0.06 |
| *Pecam1* | 0.55 | 0.93 | 1.18 | 0.95 | 2.15 | 1.03 | 1.37 | 0.15 |
| *Pf4* | 0.44 | 0.54 | 0.86 | 0.47 | 1.97 | -1.15 | 1.07 | 0.03 |
| *Pgf* | 0.65 | 0.20 | 1.53 | 3.70 | 2.36 | 18.38 | 0.83 | 2.53 |
| *PLAU(upa)* | 0.31 | 0.36 | 0.68 | 0.73 | 2.16 | 2.03 | 0.5 | 0.59 |
| *Plg* | 0.67 | ND | 0.17 | ND | -3.85 | ND | 0.77 | ND |
| *Prox1* | 0.33 | 0.51 | 0.72 | 1.52 | 2.22 | 2.95 | 0.27 | 0.51 |
| *Psf/sfpq* | 0.44 | 0.65 | 1.21 | 0.75 | 2.74 | 1.15 | 0.8 | 0.02 |
| *Pspc1* | 0.36 | 0.47 | 0.8 | 0.79 | 2.19 | 1.68 | 0.43 | 0.03 |
| *Serpinf1* | 0.31 | 0.12 | 1.79 | 0.89 | 5.7 | 7.61 | 2.05 | 0.95 |
| *Sphk1* | 0.58 | 0.63 | 0.45 | 2.32 | -1.30 | 3.65 | 0.12 | 1.09 |
| *Tek* | 0.22 | 0.44 | 0.54 | 1.15 | 2.43 | 2.62 | 0.95 | 0.24 |
| *Tgfa* | 0.85 | 0.43 | 2.09 | 1.03 | 2.45 | 2.40 | 0.31 | 1.00 |
| *Tgfb1* | 0.29 | 0.52 | 0.74 | 0.57 | 2.52 | 1.08 | 0.31 | 0.03 |
| *Tgfb2* | 0.3 | 0.63 | 0.72 | 0.52 | 2.39 | -1.22 | 0.39 | 0.04 |
| *Tgfbr1* | 0.44 | 0.52 | 0.81 | 0.51 | 1.86 | -1.02 | 0.57 | 0.02 |
| *Thbs1* | 0.31 | 0.48 | 0.62 | 0.38 | 2.03 | -1.27 | 0.3 | 0.23 |
| *Thbs2* | 0.35 | 0.47 | 0.7 | 0.67 | 1.98 | 1.43 | 0.28 | 0.01 |
| *Timp1* | 0.27 | 0.46 | 0.63 | 0.43 | 2.36 | -1.07 | 0.34 | 0.15 |
| *Timp2* | 0.27 | 0.52 | 0.69 | 0.90 | 2.55 | 1.73 | 0.42 | 0.04 |
| *Timp3* | 0.22 | 0.57 | 0.44 | 0.77 | 1.95 | 1.35 | 0.31 | 0.02 |
| *Vash1* | - | 0.33 | - | 2.28 | - | 6.86 | - | 4.27 |
| *Vegfa* | 1.82 | 2.09 | 5.38 | 2.28 | 2.95 | 1.09 | 0.7 | 0.04 |
| *Vegfb* | 2.94 | 0.59 | 8.39 | 1.40 | 2.85 | 2.37 | 0.43 | 0.07 |
| *Vegfc* | 1.05 | - | ND | - | ND | - | ND | - |
| *Vegfd* | 0.23 | 0.48 | 0.56 | 0.85 | 2.44 | 1.78 | 0.96 | 0.23 |
| *Vegfr2 (kdr)* | 0.25 | 0.37 | 0.58 | 0.57 | 2.29 | 1.55 | 0.55 | 0.15 |

**Hypoxia: 24 h**

| **Gene name** | **Total mRNA** | **Polysome bound mRNA** | **Fold change** | **Standard deviation** |
| --- | --- | --- | --- | --- |
| *Akt1* | 1.80 | 5.69 | 3.17 | 0.22 |
| *Ang* | 1.88 | 2.38 | 1.26 | 0.15 |
| *Angpt1* | 1.17 | 2.62 | 2.24 | 0.08 |
| *Angptl4* | ND | ND | ND | ND |
| *Anpep* | 4.68 | ND | ND | ND |
| *Apelin* | 31.49 | ND | ND | ND |
| *Aplnnr* | ND | ND | ND | ND |
| *Atp2a2* | 3.34 | 2.73 | -1.22 | 0.12 |
| *Bai1* | 1.39 | ND | ND | ND |
| *Ccl2* | ND | ND | ND | ND |
| *Ccl21a* | ND | 0.83 | ND | ND |
| *Col18a1* | 4.29 | 12.60 | 2.94 | 0.27 |
| *Col4a3* | 5.14 | 9.53 | 1.85 | 0.21 |
| *Ctgf* | 1.83 | 2.75 | 1.50 | 0.07 |
| *Cxcl1* | 1.09 | 1.04 | -1.06 | 0.23 |
| *Cxcl10 (inp10)* | 10.42 | ND | ND | ND |
| *Cyr 61* | 4.57 | 5.52 | 1.21 | 0.12 |
| *Edn1* | 2.40 | 2.13 | -1.12 | 0.04 |
| *Efna1* | 4.02 | 2.79 | -1.44 | 0.04 |
| *Efnb2* | 2.86 | 3.03 | 1.06 | 0.07 |
| *Egf* | 2.33 | ND | ND | ND |
| *Eng* | 4.50 | ND | D | ND |
| *Ephb4* | 2.67 | 3.37 | 1.26 | 0.01 |
| *Erbb2(her2)* | 2.26 | 4.96 | 2.20 | 0.34 |
| *F3* | 3.72 | 5.21 | 1.40 | 0.06 |
| *Fgf1* | 1.00 | ND | ND | ND |
| *Fgf2* | - | - | - | - |
| *Fgfr3* | 5.01 | 9.20 | 1.84 | 0.35 |
| *Fibrillarin* | 2.13 | 2.49 | 1.17 | 0.22 |
| *Fn1* | ND | ND | ND | ND |
| *Hif1a* | 1.24 | 2.75 | 2.21 | 0.21 |
| *Hif2a* | 1.56 | ND | ND | ND |
| *Hnrnpm* | 1.25 | 7.91 | 6.34 | 0.76 |
| *Hpse* | 17.68 | 7.70 | -2.30 | 0.07 |
| *Id1* | 1.75 | ND | ND | ND |
| *Ifna1* | ND | ND | ND | ND |
| *Igf1* | 1.73 | 1.42 | -1.22 | 0.07 |
| *Igf1r* | 4.65 | 4.94 | 1.06 | 0.00 |
| *Itgav* | 5.01 | 7.29 | 1.46 | 0.17 |
| *Itgb3* | 1.99 | 15.07 | 7.56 | 0.49 |
| *Jag1* | ND | ND | ND | ND |
| *Mdk* | 2.08 | ND | ND | ND |
| *Mmp14* | 3.04 | 4.42 | 1.45 | 0.17 |
| *Mmp2* | 2.88 | ND | ND | ND |
| *Neat-1* | 3.20 | 3.44 | 1.08 | 0.17 |
| *Nos3* | ND | ND | ND | ND |
| *Nrp1* | 3.04 | 2.84 | -1.07 | 0.12 |
| *Nrp2* | 4.42 | 5.27 | 1.19 | 0.07 |
| *P54nrb* | 2.55 | 7.61 | 2.99 | 0.08 |
| *Pai-1* | 18.19 | ND | ND | ND |
| *Pdgfa* | 2.90 | 10.05 | 3.46 | 0.34 |
| *Pecam1* | 11.37 | ND | ND | ND |
| *Pf4* | 1.16 | ND | ND | ND |
| *Pgf* | 6.82 | ND | ND | ND |
| *PLAU(upa)* | 4.42 | 2.28 | -1.94 | 0.00 |
| *Plg* | ND | ND | ND | ND |
| *Prox1* | 1.10 | 3.35 | 3.04 | 0.28 |
| *Psf/sfpq* | 0.86 | 4.59 | 5.32 | 0.05 |
| *Pspc1* | 1.23 | 1.73 | ND | ND |
| *Serpinf1* | 15.49 | ND | ND | ND |
| *Sphk1* | 6.11 | ND | ND | ND |
| *Tek* | 4.07 | 5.49 | 1.35 | 0.16 |
| *Tgfa* | 4.75 | ND | ND | ND |
| *Tgfb1* | 3.41 | 5.67 | 1.66 | 0.14 |
| *Tgfb2* | 4.72 | 1.80 | -2.63 | 0.06 |
| *Tgfbr1* | 2.08 | 2.37 | 1.14 | 0.23 |
| *Thbs1* | 5.35 | 4.11 | -1.30 | 0.10 |
| *Thbs2* | 3.77 | 3.29 | -1.15 | 0.01 |
| *Timp1* | 2.75 | 2.05 | -1.34 | 0.12 |
| *Timp2* | 2.12 | 1.94 | -1.09 | 0.14 |
| *Timp3* | 5.20 | ND | ND | ND |
| *Vash1* | 7.43 | ND | ND | ND |
| *Vegfa* | 8.15 | 14.73 | 1.81 | 0.19 |
| *Vegfb* | 1.94 | 6.41 | 3.31 | 0.34 |
| *Vegfc* | - | - | - | - |
| *Vegfd* | 2.77 | ND | ND | ND |
| *Vegfr2 (kdr)* | 3.04 | 2.28 | -1.33 | 0.13 |
