# Supplementary material for: Vasohibin1, a new mouse cardiomyocyte IRES trans-acting factor that regulates translation in early hypoxia
Source: eLife. 2019 Dec 9;8:e50094. doi: 10.7554/eLife.50094 (PMC6946400; doi:10.7554/eLife.50094)
Supplement: Supplementary file 3. — Luciferase activity values and IRES activities corresponding to the experiments presented in Figure 4. (A) Kinetics of FGF1 IRES activity from 30 min to 24 hr. (B–I) Activities of the different IRES after 4 hr, 8 hr and 24 hr of hypoxia. (J) Negative control with a lentivector containing a hairpin (no IRES) between the two luciferase cistrons. For each IRES and for each time, nine biological replicates were performed (n = 9). Each biological replicate corresponds to the mean of three technical replicates. Means, standard deviations (SD) and Mann-Whitney P values comparing IRES activities in hypoxia and in normoxia were calculated. The means are reported in the histograms shown in Figure 4. P-value significance is indicated: *p<0.05, **p<0.01, ***<0.001, ****p<0.0001. [file elife-50094-supp3.docx]

**Hantelys et al, Supplementary File 3**

**A/ Kinetics of FGF1 IRES activity in hypoxia (30 min to 24 h)**

**Normoxia Hypoxia**

**
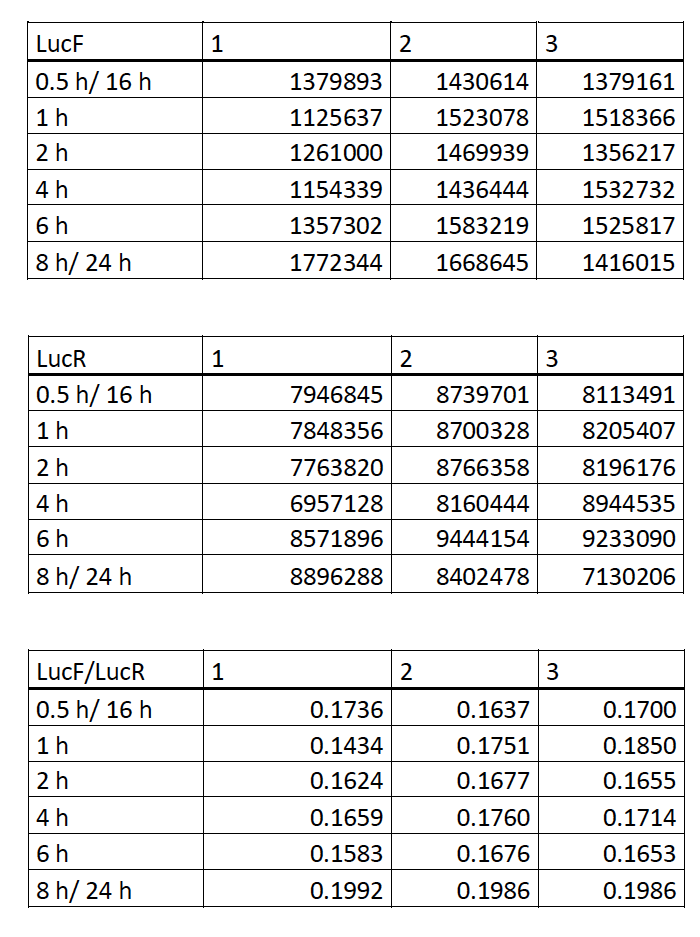

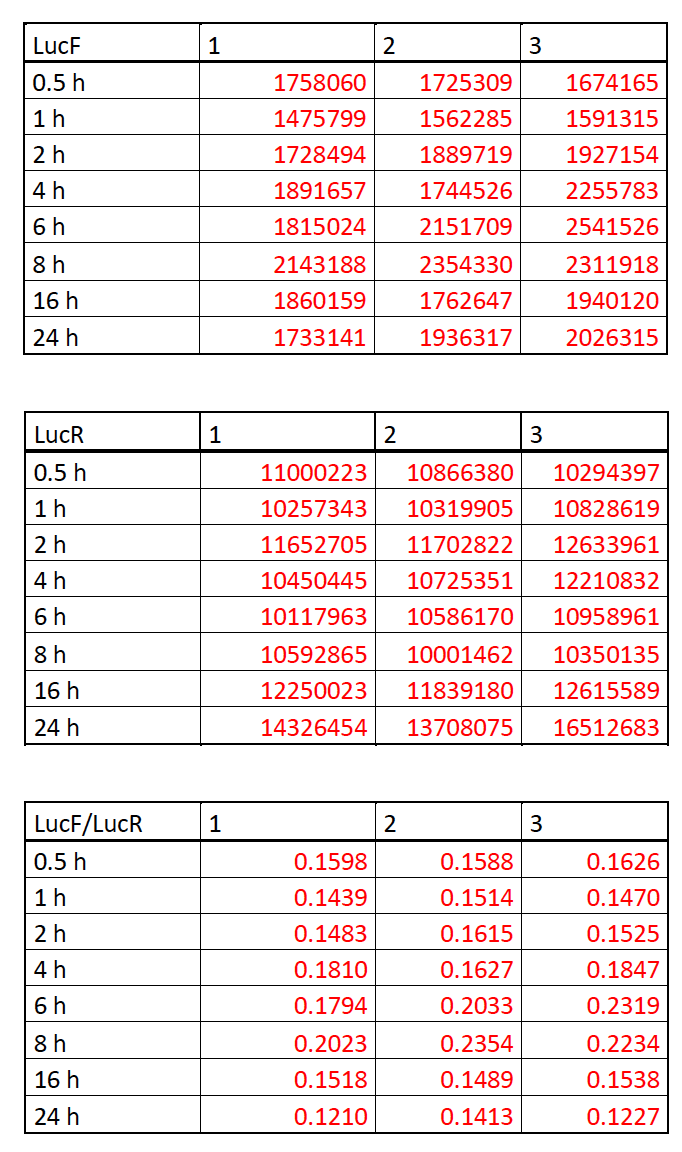
**

**IRES activity**

| Hypoxia time (h) | LucF/LucR  Mean | SD | **IRES activity: LucF/LucR *100** | SD | Mann-Whitney  test  P Value | Significance |
| --- | --- | --- | --- | --- | --- | --- |
| 0 (n=18) | 0.1726 | 0.0131 | 17.26 | 1.31 |  |  |
| 0.5 (n=3) | 0.1604 | 0.0020 | 16.04 | 0.20 | 0.0444 | * |
| 1 (n=3) | 0.1474 | 0.0038 | 14.74 | 0.38 | 0.0098 | ** |
| 2 (n=3) | 0.1541 | 0.0067 | 15.41 | 0.67 | 0.0158 | ** |
| 4 (n=3) | 0.1761 | 0.0118 | 17.61 | 1.18 | 0.7308 |  |
| 6 (n=3) | 0.2049 | 0.0263 | 20.49 | 2.63 | 0.0165 | * |
| 8 (n=3) | 0.2204 | 0.0167 | 22.04 | 1.67 | 0.0015 | ** |
| 16 (n=3) | 0.1515 | 0.0025 | 15.15 | 0.25 | 0.0098 | ** |
| 24 (n=3) | 0.1283 | 0.0112 | 12.83 | 1.12 | 0.0015 | ** |
|  |  |  |  |  |  | *= p<0.05 |
|  |  |  |  |  |  | **=p<0.01 |
|  |  |  |  |  |  |  |

**B/ FGF1 IRES**

**LucF**

|  |  | **Biological replicates** | | | | | | | | | | | | | |  |  |  |  |  |  |
| --- | --- | --- | --- | --- | --- | --- | --- | --- | --- | --- | --- | --- | --- | --- | --- | --- | --- | --- | --- | --- | --- |
| Time | Condition | 1 | 2 | | 3 | 4 | | 5 | | 6 | | 7 | 8 | 9 | |  |  |  |  |  |  |
| 4h | Normoxia | 77 310 | 91 175 | | 63 445 | 52 728 | | 58 279 | | 56 285 | | 59 485 | 53 665 | 53 223 | |  |  |  |  |  |  |
|  | Hypoxia | 248 088 | 192 876 | | 303 300 | 138 626 | | 171 997 | | 152 394 | | 110 552 | 157 006 | 149 316 | |  |  |  |  |  |  |
| 8h | Normoxia | 3 721 | 5 041 | | 2 401 | 72 622 | | 72 505 | | 73 578 | | 121 992 | 112 068 | 132 044 | |  |  |  |  |  |  |
|  | Hypoxia | 10 760 | 12 896 | | 8 624 | 70 529 | | 72 640 | | 33 059 | | 118 486 | 109 078 | 112 198 | |  |  |  |  |  |  |
| 24h | Normoxia | 231 071 | 198 199 | | 263 943 | 208 454 | | 220 996 | | 210 670 | | 226 971 | 199 587 | 262 787 | |  |  |  |  |  |  |
|  | Hypoxia | 138 279 | 153 534 | | 123 025 | 100 770 | | 113 450 | | 99 785 | | 105 268 | 70 597 | 90 603 | |  |  |  |  |  |  |
| **LucR** | | | |  | | |  | |  | |  | | | |  | |  |  |  |  |  |
|  |  | **Biological replicates** | | | | | | | | | | | | | |  |  |  |  |  |  |
| Time | Condition | 1 | 2 | | 3 | 4 | | 5 | | 6 | | 7 | 8 | 9 | |  |  |  |  |  |  |
| 4h | Normoxia | 604 174 | 507 891 | | 700 457 | 452 118 | | 345 763 | | 472 356 | | 495 500 | 529 252 | 547 526 | |  |  |  |  |  |  |
|  | Hypoxia | 996 143 | 899 215 | | 1 093 070 | 714 978 | | 915 991 | | 757 182 | | 754 720 | 974 680 | 917 680 | |  |  |  |  |  |  |
| 8h | Normoxia | 12 580 | 13 628 | | 11 532 | 700 579 | | 710 733 | | 712 400 | | 912 286 | 860 603 | 855 579 | |  |  |  |  |  |  |
|  | Hypoxia | 35 746 | 46 304 | | 25 188 | 631 364 | | 634 140 | | 633 140 | | 826 664 | 827 015 | 822 262 | |  |  |  |  |  |  |
| 24h | Normoxia | 1 010 280 | 1 134 589 | | 885 971 | 1 021 135 | | 1 036 550 | | 1 020 270 | | 956 252 | 896 252 | 789 825 | |  |  |  |  |  |  |
|  | Hypoxia | 662 414 | 716 741 | | 608 087 | 519 324 | | 514 720 | | 769 988 | | 455 239 | 386 245 | 351 256 | |  |  |  |  |  |  |

| **LucF/LucR** | |  | | | |  | | |  |  |  | |  |  |  |  |
| --- | --- | --- | --- | --- | --- | --- | --- | --- | --- | --- | --- | --- | --- | --- | --- | --- |
|  |  | | **Biological replicates** | | | | | | | | | | | | | |
| Time | Condition | | 1 | 2 | 3 | | 4 | 5 | | | | 6 | | 7 | 8 | 9 |
| 4h | Normoxia | | 0.1280 | 0.1795 | 0.0906 | | 0.1166 | 0.1686 | | | | 0.1192 | | 0.1201 | 0.1014 | 0.0972 |
|  | Hypoxia | | 0.2490 | 0.2145 | 0.2775 | | 0.1939 | 0.1878 | | | | 0.2013 | | 0.1465 | 0.1611 | 0.1627 |
| 8h | Normoxia | | 0.2958 | 0.3699 | 0.2082 | | 0.1037 | 0.1020 | | | | 0.1033 | | 0.1337 | 0.1302 | 0.1543 |
|  | Hypoxia | | 0.3010 | 0.2785 | 0.3424 | | 0.1117 | 0.1145 | | | | 0.0522 | | 0.1433 | 0.1319 | 0.1365 |
| 24h | Normoxia | | 0.2287 | 0.1747 | 0.2979 | | 0.2041 | 0.2132 | | | | 0.2065 | | 0.2374 | 0.2227 | 0.3327 |
|  | Hypoxia | | 0.2338 | 0.1478 | 0.1675 | | 0.1974 | 0.2435 | | | | 0.1885 | | 0.2312 | 0.1828 | 0.2579 |

**IRES activity (AU : LucF/LuR x 100)**

| Time | Condition | Total mean | SD | H/N ratio | M-W test  P value | Significance |
| --- | --- | --- | --- | --- | --- | --- |
| 4h | Normoxia | 12.46 | 3.07 |  | 0.0012 | ** |
|  | Hypoxia | 19.94 | 4.27 | **1.60** |  |  |
| 8h | Normoxia | 17.79 | 9.57 |  | 0.8633 |  |
|  | Hypoxia | 17.91 | 10.10 | 1.01 |  |  |
| 24h | Normoxia | 23.53 | 4.95 |  | 0.2581 |  |
|  | Hypoxia | 20.46 | 3.56 | 0.87 |  |  |

**C/ FGF2 IRES**

**LucF**

|  | | | | | | | | | | | |  |  |  |  |  |  |  |  |  |  |
| --- | --- | --- | --- | --- | --- | --- | --- | --- | --- | --- | --- | --- | --- | --- | --- | --- | --- | --- | --- | --- | --- |
|  |  | **Biological replicates** | | | | | | | | |  |  |  |  |  |  |  |  |  |  |  |
| Time | Condition | 1 | 2 | 3 | 4 | 5 | 6 | 7 | 8 | 9 |  |  |  |  |  |  |  |  |  |  |  |
| 4h | Normoxia | 89 743 | 98 302 | 91 878 | 104 059 | 102 906 | 102 632 | 80 652 | 78 568 | 70 687 |  |  |  |  |  |  |  |  |  |  |  |
|  | Hypoxia | 109 876 | 111 252 | 98 034 | 109 548 | 111 252 | 120 034 | 85 698 | 90 565 | 84 568 |  |  |  |  |  |  |  |  |  |  |  |
| 8h | Normoxia | 146 589 | 149 931 | 129 865 | 84 185 | 90 263 | 75623 | 80 256 | 77 895 | 79 568 |  |  |  |  |  |  |  |  |  |  |  |
|  | Hypoxia | 124 357 | 130 350 | 152 345 | 85 227 | 82 565 | 78 265 | 80 268 | 82 568 | 79 635 |  |  |  |  |  |  |  |  |  |  |  |
| 24h | Normoxia | 116 853 | 120 498 | 124 567 | 226 539 | 198 256 | 161 526 | 178 258 | 170 625 | 168 262 |  |  |  |  |  |  |  |  |  |  |  |
|  | Hypoxia | 182 431 | 173 530 | 165 431 | 245 261 | 256 154 | 198 782 | 258 684 | 298 365 | 224 265 |  |  |  |  |  |  |  |  |  |  |  |
|  |  |  |  |  |  |  |  |  |  |  |  |  |  |  |  |  |  |  |  |  |  |
| **LucR** |  |  |  |  |  |  |  |  |  |  |  |  |  |  |  |  |  |  |  |  |  |
|  |  | **Biological replicates** | | | | | | | | |  |  |  |  |  |  |  |  |  |  |  |
| Time | Condition | 1 | 2 | 3 | 4 | 5 | 6 | 7 | 8 | 9 |  |  |  |  |  |  |  |  |  |  |  |
| 4h | Normoxia | 628834 | 603251 | 508251 | 686008 | 676840 | 627525 | 458252 | 398254 | 405258 |  |  |  |  |  |  |  |  |  |  |  |
|  | Hypoxia | 457353 | 502343 | 376543 | 492213 | 456687 | 475340 | 386685 | 356812 | 298568 |  |  |  |  |  |  |  |  |  |  |  |
| 8h | Normoxia | 876234 | 1012834 | 786352 | 647158 | 709937 | 655582 | 478584 | 489656 | 505362 |  |  |  |  |  |  |  |  |  |  |  |
|  | Hypoxia | 508675 | 642576 | 756243 | 592213 | 556687 | 604257 | 425542 | 398568 | 405268 |  |  |  |  |  |  |  |  |  |  |  |
| 24h | Normoxia | 576834 | 715157 | 496642 | 686695 | 677527 | 628212 | 578568 | 550268 | 562454 |  |  |  |  |  |  |  |  |  |  |  |
|  | Hypoxia | 780284 | 1173705 | 987564 | 1242151 | 1051822 | 1054542 | 978587 | 857258 | 895635 |  |  |  |  |  |  |  |  |  |  |  |

| **LucF/LucR** | |  | | | |  | | |  |  |  | |  |  |  |  |
| --- | --- | --- | --- | --- | --- | --- | --- | --- | --- | --- | --- | --- | --- | --- | --- | --- |
|  |  | | **Biological replicates** | | | | | | | | | | | | | |
| Time | Condition | | 1 | 2 | 3 | | 4 | 5 | | | | 6 | | 7 | 8 | 9 |
| 4h | Normoxia | | 0.1427 | 0.1630 | 0.1808 | | 0.1517 | 0.1520 | | | | 0.1636 | | 0.1760 | 0.1973 | 0.1744 |
|  | Hypoxia | | 0.2402 | 0.2215 | 0.2604 | | 0.2226 | 0.2436 | | | | 0.2525 | | 0.2216 | 0.2538 | 0.2832 |
| 8h | Normoxia | | 0.1673 | 0.1480 | 0.1651 | | 0.1301 | 0.1271 | | | | 0.1154 | | 0.1677 | 0.1591 | 0.1574 |
|  | Hypoxia | | 0.2445 | 0.2029 | 0.2014 | | 0.1439 | 0.1483 | | | | 0.1295 | | 0.1886 | 0.2072 | 0.1965 |
| 24h | Normoxia | | 0.2026 | 0.1685 | 0.2508 | | 0.3299 | 0.2926 | | | | 0.2571 | | 0.3081 | 0.3101 | 0.2992 |
|  | Hypoxia | | 0.2338 | 0.1478 | 0.1675 | | 0.1974 | 0.2435 | | | | 0.1885 | | 0.2643 | 0.3480 | 0.2504 |

**IRES activity (AU : LucF/LuR x 100)**

| Time | Condition | Total mean | SD | H/N ratio | M-W test  P value | Significance |
| --- | --- | --- | --- | --- | --- | --- |
| 4h | Normoxia | 16.68 | 1.70 |  | <0.0001 | **** |
|  | Hypoxia | 24.44 | 2.08 | **1.46** |  |  |
| 8h | Normoxia | 14.86 | 1 .96 |  | 0.0503 | ns |
|  | Hypoxia | 18.48 | 3.69 | 1.24 |  |  |
| 24h | Normoxia | 26.88 | 5.41 |  | 0.0939 | ns |
|  | Hypoxia | 22.68 | 6.03 | 0.84 |  |  |

**D/ VEGFA IRES a**

| **LucF** |  |  | | | | |  | |  | |  | |  |  |  | |  |  | |
| --- | --- | --- | --- | --- | --- | --- | --- | --- | --- | --- | --- | --- | --- | --- | --- | --- | --- | --- | --- |
|  |  |  | | | | |  | |  | |  | |  |  |  | |  |  | |
|  |  | **Biological replicates** | | | | | | | | | | | | | | | | |  |
| Time | Condition | 1 | 2 | 3 | 4 | 5 | | 6 | | 7 | | 8 | | | | 9 | | |  |
| 4h | Normoxia | 106 987 | 7 470 | 80 384 | 80 568 | 75 356 | | 78 658 | | 68 256 | | 60 658 | | | | 58 698 | | |  |
|  | Hypoxia | 198 708 | 10 543 | 112 982 | 187 525 | 175 862 | | 168 568 | | 105 364 | | 121 231 | | | | 135 682 | | |  |
| 8h | Normoxia | 140 987 | 13 381 | 229 805 | 100 578 | 96 235 | | 97268 | | 230 264 | | 214 235 | | | | 221 658 | | |  |
|  | Hypoxia | 291 799 | 15 874 | 217 091 | 186 568 | 202 525 | | 164 570 | | 402 562 | | 380 256 | | | | 351 214 | | |  |
| 24h | Normoxia | 95 467 | 11 501 | 66 548 | 102 540 | 110 402 | | 98 758 | | 125 214 | | 123 214 | | | | 110 254 | | |  |
|  | Hypoxia | 43 998 | 3 278 | 18 093 | 60 568 | 55 845 | | 68 025 | | 70 658 | | 65 252 | | | | 64 154 | | |  |
|  |  |  |  |  |  |  | |  | |  | |  | | | |  | | |  |
| **LucR** |  |  |  |  |  |  | |  | |  | |  | | | |  | | |  |
|  |  |  |  |  |  |  | |  | |  | |  | | | |  | | |  |
|  |  | **Biological replicates** | | | | | | | | | | | | | | | | |  |
| Time | Condition | 1 | 2 | 3 | 4 | 5 | | 6 | | 7 | | 8 | | | | 9 | | |  |
| 4h | Normoxia | 557 863 | 620 150 | 654 367 | 512 362 | 458 685 | | 398 568 | | 369 568 | | 398 658 | | | | 405 235 | | |  |
|  | Hypoxia | 1 387 607 | 1 255 594 | 987 656 | 985 365 | 987 878 | | 865 258 | | 875 698 | | 870 258 | | | | 865 234 | | |  |
| 8h | Normoxia | 1 098 767 | 1 618 781 | 1 579 760 | 758 652 | 684 594 | | 705635 | | 656 265 | | 652 485 | | | | 698 584 | | |  |
|  | Hypoxia | 1 465 480 | 1 285 249 | 1 354 320 | 1 025 268 | 985 698 | | 1 014 254 | | 878 258 | | 852 012 | | | | 878 565 | | |  |
| 24h | Normoxia | 1 246 587 | 1 620 876 | 1 674 890 | 878 985 | 985 635 | | 868 457 | | 758 587 | | 765 625 | | | | 720 258 | | |  |
|  | Hypoxia | 1 054 689 | 879 823 | 987 698 | 1 121 245 | 1 002 568 | | 987 685 | | 975 685 | | 985 625 | | | | 945 252 | | |  |

| **LucF/LucR** | |  | | | |  | | |  |  |  | |  |  |  |  |
| --- | --- | --- | --- | --- | --- | --- | --- | --- | --- | --- | --- | --- | --- | --- | --- | --- |
|  |  | | **Biological replicates** | | | | | | | | | | | | | |
| Time | Condition | | 1 | 2 | 3 | | 4 | 5 | | | | 6 | | 7 | 8 | 9 |
| 4h | Normoxia | | 0.1918 | 0.0120 | 0.1228 | | 0.1572 | 0.1643 | | | | 0.1974 | | 0.1847 | 0.1522 | 0.1448 |
|  | Hypoxia | | 0.1432 | 0.0084 | 0.1144 | | 0.1903 | 0.1780 | | | | 0.1948 | | 0.1203 | 0.1393 | 0.1568 |
| 8h | Normoxia | | 0.1283 | 0.0083 | 0.1455 | | 0.1326 | 0.1406 | | | | 0.1378 | | 0.3509 | 0.3283 | 0.3173 |
|  | Hypoxia | | 0.1991 | 0.0124 | 0.1603 | | 0.1820 | 0.2055 | | | | 0.1623 | | 0.4584 | 0.4463 | 0.3998 |
| 24h | Normoxia | | 0.0766 | 0.0071 | 0.0397 | | 0.1167 | 0.1120 | | | | 0.1137 | | 0.1651 | 0.1609 | 0.1531 |
|  | Hypoxia | | 0.0417 | 0.0037 | 0.0183 | | 0.0540 | 0.0557 | | | | 0.0689 | | 0.0724 | 0.0662 | 0.0679 |

**IRES activity (AU : LucF/LuR x 100)**

| Time | Condition | Total mean | SD | H/N ratio | M-W test  P value | Significance |
| --- | --- | --- | --- | --- | --- | --- |
| 4h | Normoxia | 14.75 | 5.61 |  | 0.4363 | ns |
|  | Hypoxia | 13.84 | 5.66 | 0.94 |  |  |
| 8h | Normoxia | 18.77 | 11.64 |  | 0.1359 | ns |
|  | Hypoxia | 24.73 | 15.24 | **1.32** |  |  |
| 24h | Normoxia | 10.50 | 5.47 |  | 0 .0244 | * |
|  | Hypoxia | 4.99 | 2.42 | **0.48** |  |  |

**E/ VEGFA IRES b**

| **LucF** | | |  | | |  | |  |  | |  | |  | |  | |  | |  | |  | |
| --- | --- | --- | --- | --- | --- | --- | --- | --- | --- | --- | --- | --- | --- | --- | --- | --- | --- | --- | --- | --- | --- | --- |
|  | | |  | | |  | |  |  | |  | |  | |  | |  | |  | |  | |
|  |  | | | **Biological replicates** | | | | | | | | | | | | | | | | | |  |
| Time | | Condition | | 1 | 2 | | 3 | | | 4 | | 5 | | 6 | | 7 | | 8 | | 9 | |  |
| 4h | | Normoxia | | 140 874 | 98 149 | | 100 854 | | | 102 368 | | 112 327 | | 106 258 | | 104 241 | | 124 251 | | 114 212 | |  |
|  |  | Hypoxia | | 100 976 | 91 883 | | 93 743 | | | 86 358 | | 87 652 | | 85 365 | | 80 242 | | 78 658 | | 81 542 | |  |
| 8h | | Normoxia | | 159 873 | 208 754 | | 170 983 | | | 130 254 | | 112 584 | | 112 547 | | 124 212 | | 110 226 | | 102 265 | |  |
|  |  | Hypoxia | | 378 983 | 513 871 | | 389 750 | | | 298 654 | | 305 265 | | 300 245 | | 310 245 | | 302 142 | | 298 265 | |  |
| 24h | | Normoxia | | 499 864 | 537 099 | | 556 098 | | | 498 567 | | 465 856 | | 475 554 | | 487 568 | | 452 028 | | 435 982 | |  |
|  |  | Hypoxia | | 350 980 | 439 788 | | 345 230 | | | 345 268 | | 344 568 | | 324 265 | | 285 475 | | 284 658 | | 235 268 | |  |
|  | |  | |  |  | |  | | |  | |  | |  | |  | |  | |  | |  |
| **LucR** | |  | |  |  | |  | | |  | |  | |  | |  | |  | |  | |  |
|  | |  | |  |  | |  | | |  | |  | |  | |  | |  | |  | |  |
|  |  | | | **Biological replicates** | | | | | | | | | | | | | | | | | |  |
| Time | | Condition | | 1 | 2 | | 3 | | | 4 | | 5 | | 6 | | 7 | | 8 | | 9 | |  |
| 4h | | Normoxia | | 1 087 650 | 2 019 783 | | 1 876 580 | | | 986 256 | | 968 586 | | 987 584 | | 876 548 | | 857 625 | | 985 562 | |  |
|  |  | Hypoxia | | 2 098 750 | 2 993 873 | | 3 542 712 | | | 1 987 584 | | 1 876 258 | | 1 785 625 | | 1 685 265 | | 1 457 235 | | 1 471 457 | |  |
| 8h | | Normoxia | | 2 178 659 | 2 269 092 | | 2 157 890 | | | 1 587 568 | | 1 658 258 | | 1 785 547 | | 1 325 328 | | 1 245 258 | | 1 475 682 | |  |
|  |  | Hypoxia | | 2 765 909 | 3 127 386 | | 2 987 652 | | | 2 258 586 | | 2 457 268 | | 2 358 625 | | 2 425 241 | | 2 574 625 | | 2 145 258 | |  |
| 24h | | Normoxia | | 3 987 600 | 3 794 801 | | 3 654 780 | | | 3 685 658 | | 3 545 658 | | 3 365 245 | | 3 258 654 | | 3 214 651 | | 3 254 478 | |  |
|  |  | Hypoxia | | 3 265 890 | 3 614 913 | | 2 764 579 | | | 2 998 586 | | 3 058 248 | | 3 124 025 | | 3 021 214 | | 2 875 625 | | 2 874 246 | |  |

| **LucF/LucR** | |  | | | |  | | |  |  |  | |  |  |  |  |
| --- | --- | --- | --- | --- | --- | --- | --- | --- | --- | --- | --- | --- | --- | --- | --- | --- |
|  |  | | **Biological replicates** | | | | | | | | | | | | | |
| Time | Condition | | 1 | 2 | 3 | | 4 | 5 | | | | 6 | | 7 | 8 | 9 |
| 4h | Normoxia | | 0.1295 | 0.0486 | 0.0537 | | 0.1038 | 0.1160 | | | | 0.1076 | | 0.1189 | 0.1449 | 0.1159 |
|  | Hypoxia | | 0.0481 | 0.0307 | 0.0265 | | 0.0434 | 0.0467 | | | | 0.0478 | | 0.0476 | 0.0540 | 0.0554 |
| 8h | Normoxia | | 0.0734 | 0.0920 | 0.0792 | | 0.0820 | 0.0679 | | | | 0.0630 | | 0.0937 | 0.0885 | 0.0693 |
|  | Hypoxia | | 0.1370 | 0.1643 | 0.1305 | | 0.1322 | 0.1242 | | | | 0.1273 | | 0.1279 | 0.1174 | 0.1390 |
| 24h | Normoxia | | 0.1254 | 0.1415 | 0.1522 | | 0.1353 | 0.1314 | | | | 0.1413 | | 0.1496 | 0.1406 | 0.1340 |
|  | Hypoxia | | 0.1075 | 0.1217 | 0.1249 | | 0.1151 | 0.1127 | | | | 0.1038 | | 0.0945 | 0.0990 | 0.0819 |

**IRES activity (AU : LucF/LuR x 100)**

| Time | Condition | Total mean | SD | H/N ratio | M-W test  P value | Significance |
| --- | --- | --- | --- | --- | --- | --- |
| 4h | Normoxia | 10.43 | 3.25 |  | 0.0005 | *** |
|  | Hypoxia | 4.45 | 0.98 | **0.43** |  |  |
| 8h | Normoxia | 7.88 | 1.11 |  | <0.0001 | **** |
|  | Hypoxia | 13.33 | 1.33 | **1.69** |  |  |
| 24h | Normoxia | 13.90 | 0.85 |  | <0.0001 | **** |
|  | Hypoxia | 10.68 | 1.37 | **0.77** |  |  |

**F/ VEGFC IRES**

| **LucF** | |  |  |  |  |  |  |  |  |  |  |
| --- | --- | --- | --- | --- | --- | --- | --- | --- | --- | --- | --- |
|  | |  |  |  |  |  |  |  |  |  |  |
|  |  | | **Biological replicates** | | | | | | | | |
| Time | | Condition | 1 | 2 | 3 | 4 | 5 | 6 | 7 | 8 | 9 |
| 4h | | Normoxia | 17 645 | 16 237 | 15 672 | 15 245 | 14 582 | 13 258 | 18 750 | 16 985 | 19 754 |
|  |  | Hypoxia | 21 673 | 18 377 | 17 834 | 19 875 | 18 625 | 17 485 | 17 258 | 16 238 | 14 587 |
| 8h | | Normoxia | 21 673 | 18 377 | 17 834 | 20 235 | 20 146 | 19 826 | 16 987 | 17 258 | 16 784 |
|  |  | Hypoxia | 27 742 | 18 374 | 19 875 | 26 587 | 21 457 | 20 358 | 30 268 | 31 245 | 30 216 |
| 24h | | Normoxia | 23 451 | 21 422 | 22 765 | 21 568 | 20 148 | 18 759 | 19 867 | 20 235 | 28 220 |
|  |  | Hypoxia | 30 194 | 22 544 | 26 876 | 28 568 | 27 532 | 26 352 | 19 287 | 24 568 | 22 586 |
|  | |  |  |  |  |  |  |  |  |  |  |
| **LucR** | |  |  |  |  |  |  |  |  |  |  |
|  | |  |  |  |  |  |  |  |  |  |  |
|  |  | | **Biological replicates** | | | | | | | | |
| Time | | Condition | 1 | 2 | 3 | 4 | 5 | 6 | 7 | 8 | 9 |
| 4h | | Normoxia | 287 654 | 316 145 | 365 786 | 195 685 | 185 247 | 182 457 | 205 236 | 204 325 | 210 214 |
|  |  | Hypoxia | 302 234 | 354 485 | 345 290 | 298 538 | 290 265 | 284 242 | 280 142 | 270 268 | 262 874 |
| 8h | | Normoxia | 425 763 | 455 482 | 480 013 | 390 584 | 382 546 | 367 856 | 356 258 | 321 252 | 384 276 |
|  |  | Hypoxia | 403 294 | 341 909 | 375 634 | 390 268 | 352 632 | 342 268 | 410 226 | 401 236 | 410 256 |
| 24h | | Normoxia | 420 632 | 391 071 | 375 100 | 410 214 | 402 158 | 402 387 | 398 268 | 396 216 | 381 568 |
|  |  | Hypoxia | 452 093 | 342 612 | 410 973 | 402 358 | 412 586 | 398 574 | 398 265 | 396 246 | 362 142 |

| **LucF/LucR** | |  | | | |  | | |  |  |  | |  |  |  |  |
| --- | --- | --- | --- | --- | --- | --- | --- | --- | --- | --- | --- | --- | --- | --- | --- | --- |
|  |  | | **Biological replicates** | | | | | | | | | | | | | |
| Time | Condition | | 1 | 2 | 3 | | 4 | 5 | | | | 6 | | 7 | 8 | 9 |
| 4h | Normoxia | | 0.0613 | 0.0514 | 0.0428 | | 0.0779 | 0.0787 | | | | 0.0727 | | 0.0914 | 0.0831 | 0.0940 |
|  | Hypoxia | | 0.0717 | 0.0518 | 0.0516 | | 0.0666 | 0.0642 | | | | 0.0615 | | 0.0616 | 0.0601 | 0.0555 |
| 8h | Normoxia | | 0.0509 | 0.0403 | 0.0372 | | 0.0518 | 0.0527 | | | | 0.0539 | | 0.0477 | 0.0537 | 0.0437 |
|  | Hypoxia | | 0.0688 | 0.0537 | 0.0529 | | 0.0681 | 0.0608 | | | | 0.0595 | | 0.0738 | 0.0779 | 0.0737 |
| 24h | Normoxia | | 0.0558 | 0.0548 | 0.0607 | | 0.0526 | 0.0501 | | | | 0.0466 | | 0.0499 | 0.0511 | 0.0740 |
|  | Hypoxia | | 0.0668 | 0.0658 | 0.0654 | | 0.0710 | 0.0667 | | | | 0.0661 | | 0.0484 | 0.0620 | 0.0624 |

**IRES activity (AU : LucF/LuR x 100)**

| Time | Condition | Total mean | SD | H/N ratio | M-W test  P value | Significance |
| --- | --- | --- | --- | --- | --- | --- |
| 4h | Normoxia | 7.26 | 1.75 |  | 0.1359 | ns |
|  | Hypoxia | 6.05 | 0.67 | 0.83 |  |  |
| 8h | Normoxia | 4.80 | 0.62 |  | 0.0003 | *** |
|  | Hypoxia | 6.55 | 0.91 | **1.36** |  |  |
| 24h | Normoxia | 5.50 | 0.82 |  | 0.0315 | * |
|  | Hypoxia | 6.38 | 0.64 | **1.16** |  |  |

**G/ VEGFD IRES**

| **LucF** | |  |  |  |  |  |  |  |  |  |  |
| --- | --- | --- | --- | --- | --- | --- | --- | --- | --- | --- | --- |
|  | |  |  |  |  |  |  |  |  |  |  |
|  |  | | **Biological replicates** | | | | | | | | |
| Time | | Condition | 1 | 2 | 3 | 4 | 5 | 6 | 7 | 8 | 9 |
| 4h | | Normoxia | 1 123 453 | 1 092 536 | 1 242 342 | 125 682 | 123 122 | 120 268 | 1 252 025 | 985 986 | 1 025 254 |
|  |  | Hypoxia | 1 087 648 | 1 129 996 | 1 250 434 | 110 236 | 112 025 | 99 856 | 1 452 638 | 1 258 326 | 1 425 325 |
| 8h | | Normoxia | 899 766 | 837 544 | 657 889 | 89 526 | 75 862 | 78 588 | 1 418 588 | 1 325 682 | 1 312 022 |
|  |  | Hypoxia | 1 141 352 | 977 778 | 855 973 | 112 252 | 85 211 | 92 252 | 1 912 212 | 1 858 562 | 1 757 552 |
| 24h | | Normoxia | 1 187 460 | 1 382 536 | 1 289 532 | 147 214 | 132 250 | 145 258 | 1 547 252 | 1 457 258 | 1 325 206 |
|  |  | Hypoxia | 521 596 | 706 269 | 506 468 | 50 231 | 48 215 | 47 528 | 485 625 | 465 258 | 475 528 |
|  | |  |  |  |  |  |  |  |  |  |  |
| **LucR** | |  |  |  |  |  |  |  |  |  |  |
|  | |  |  |  |  |  |  |  |  |  |  |
|  |  | | **Biological replicates** | | | | | | | | |
| Time | | Condition | 1 | 2 | 3 | 4 | 5 | 6 | 7 | 8 | 9 |
| 4h | | Normoxia | 11 987 690 | 12 069 833 | 12 069 833 | 1 265 226 | 1 125 268 | 1 125 363 | 10 252 682 | 9 253 258 | 11 220 214 |
|  |  | Hypoxia | 10 657 845 | 12 059 630 | 11 656 849 | 1 025 236 | 989 265 | 1 125 236 | 11 212 241 | 13 258 233 | 12 258 225 |
| 8h | | Normoxia | 13 799 872 | 14 349 216 | 12 764 891 | 1 325 662 | 1 685 236 | 1 256 364 | 12 452 120 | 11 572 582 | 11 457 582 |
|  |  | Hypoxia | 11 678 964 | 10 969 410 | 12 345 654 | 1 152 635 | 1 232 568 | 1 152 332 | 10 214 785 | 9 865 236 | 11 525 852 |
| 24h | | Normoxia | 14 908 768 | 16 101 138 | 15 779 780 | 1 658 235 | 1 751 820 | 1 256 250 | 10 475 685 | 11 452 232 | 10 254 362 |
|  |  | Hypoxia | 8 765 789 | 9 428 743 | 9 076 890 | 895 652 | 952 325 | 875 252 | 9 258 352 | 8 562 265 | 8 956 825 |

| **LucF/LucR** | |  | | | |  | | |  |  |  | |  |  |  |  |
| --- | --- | --- | --- | --- | --- | --- | --- | --- | --- | --- | --- | --- | --- | --- | --- | --- |
|  |  | | **Biological replicates** | | | | | | | | | | | | | |
| Time | Condition | | 1 | 2 | 3 | | 4 | 5 | | | | 6 | | 7 | 8 | 9 |
| 4h | Normoxia | | 0.0937 | 0.0905 | 0.1029 | | 0.0993 | 0.1094 | | | | 0.1069 | | 0.1221 | 0.1066 | 0.0914 |
|  | Hypoxia | | 0.1021 | 0.0937 | 0.1073 | | 0.1075 | 0.1132 | | | | 0.0887 | | 0.1296 | 0.0949 | 0.1163 |
| 8h | Normoxia | | 0.0652 | 0.0584 | 0.0515 | | 0.0675 | 0.0450 | | | | 0.0626 | | 0.1139 | 0.1146 | 0.1145 |
|  | Hypoxia | | 0.0977 | 0.0891 | 0.0693 | | 0.0974 | 0.0691 | | | | 0.0801 | | 0.1872 | 0.1884 | 0.1525 |
| 24h | Normoxia | | 0.0796 | 0.0859 | 0.0817 | | 0.0888 | 0.0755 | | | | 0.1156 | | 0.1477 | 0.1272 | 0.1292 |
|  | Hypoxia | | 0.0595 | 0.0749 | 0.0558 | | 0.0561 | 0.0506 | | | | 0.0543 | | 0.0525 | 0.0543 | 0.0531 |

**IRES activity (AU : LucF/LuR x 100)**

| Time | Condition | Total mean | SD | H/N ratio | M-W test  P value | Significance |
| --- | --- | --- | --- | --- | --- | --- |
| 4h | Normoxia | 10.25 | 1.01 |  | 0.5043 | ns |
|  | Hypoxia | 10.59 | 1.28 | **1.03** |  |  |
| 8h | Normoxia | 7.70 | 2.88 |  | 0.0503 | ns |
|  | Hypoxia | 11.45 | 4.83 | **1.49** |  |  |
| 24h | Normoxia | 10.35 | 2.67 |  | <0.0001 | **** |
|  | Hypoxia | 5.68 | 0.72 | **0.55** |  |  |

**H/ c-myc IRES**

| **LucF** | |  |  |  |  |  |  |  |  |  |  |
| --- | --- | --- | --- | --- | --- | --- | --- | --- | --- | --- | --- |
|  | |  |  |  |  |  |  |  |  |  |  |
|  |  | | **Biological replicates** | | | | | | | | |
| Time | | Condition | 1 | 2 | 3 | 4 | 5 | 6 | 7 | 8 | 9 |
| 4h | | Normoxia | 2 567 872 | 3 651 734 | 3 454 332 | 356 812 | 375 210 | 351 278 | 360 212 | 410 251 | 420 215 |
|  |  | Hypoxia | 2 029 809 | 3 147 437 | 3 256 210 | 275 895 | 265 826 | 245 628 | 310 215 | 325 230 | 312 020 |
| 8h | | Normoxia | 3 546 872 | 3 161 720 | 3 478 284 | 368 258 | 450 268 | 452 025 | 415 231 | 398 652 | 350 652 |
|  |  | Hypoxia | 3 508 730 | 2 749 546 | 2 876 921 | 325 120 | 301 245 | 298 547 | 350 655 | 316 522 | 310 222 |
| 24h | | Normoxia | 7 768 312 | 8 684 097 | 6 987 312 | 358 582 | 258 625 | 247 583 | 362 025 | 352 014 | 342 062 |
|  |  | Hypoxia | 7 513 771 | 5 782 962 | 7 981 903 | 486 250 | 460 258 | 410 258 | 658 250 | 568 260 | 487 210 |
|  | |  |  |  |  |  |  |  |  |  |  |
| **LucR** | |  |  |  |  |  |  |  |  |  |  |
|  | |  |  |  |  |  |  |  |  |  |  |
|  |  | | **Biological replicates** | | | | | | | | |
| Time | | Condition | 1 | 2 | 3 | 4 | 5 | 6 | 7 | 8 | 9 |
| 4h | | Normoxia | 29 784 420 | 27 508 124 | 26 098 324 | 3 025 325 | 2 758 632 | 2 658 985 | 2 879 258 | 2 586 258 | 2 586 258 |
|  |  | Hypoxia | 20 988 822 | 19 862 926 | 21 341 228 | 2 025 128 | 2 212 570 | 2 325 201 | 2 145 251 | 2 014 214 | 2 147 542 |
| 8h | | Normoxia | 24 900 832 | 29 630 539 | 30 983 221 | 2 856 985 | 2 140 236 | 2 562 120 | 3 125 368 | 2 582 321 | 2 836 124 |
|  |  | Hypoxia | 23 987 042 | 24 750 406 | 25 987 577 | 2 653 213 | 2 012 251 | 2 120 124 | 2 785 624 | 2 658 325 | 2 625 258 |
| 24h | | Normoxia | 36 987 908 | 35 408 692 | 23 788 902 | 3 856 658 | 4 021 522 | 4 010 698 | 4 025 215 | 4 085 368 | 4 125 214 |
|  |  | Hypoxia | 18 898 912 | 13 608 479 | 16 988 904 | 4 582 632 | 4 658 230 | 4 215 120 | 4 658 284 | 4 075 250 | 4 875 251 |

| **LucF/LucR** | |  | | | |  | | |  |  |  | |  |  |  |  |
| --- | --- | --- | --- | --- | --- | --- | --- | --- | --- | --- | --- | --- | --- | --- | --- | --- |
|  |  | | **Biological replicates** | | | | | | | | | | | | | |
| Time | Condition | | 1 | 2 | 3 | | 4 | 5 | | | | 6 | | 7 | 8 | 9 |
| 4h | Normoxia | | 0.0862 | 0.1328 | 0.1324 | | 0.1179 | 0.1360 | | | | 0.1321 | | 0.1251 | 0.1586 | 0.1625 |
|  | Hypoxia | | 0.0967 | 0.1585 | 0.1526 | | 0.1362 | 0.1201 | | | | 0.1056 | | 0.1446 | 0.1615 | 0.1453 |
| 8h | Normoxia | | 0.1424 | 0.1067 | 0.1123 | | 0.1289 | 0.2104 | | | | 0.1764 | | 0.1329 | 0.1544 | 0.1236 |
|  | Hypoxia | | 0.1463 | 0.1111 | 0.1107 | | 0.1225 | 0.1497 | | | | 0.1408 | | 0.1259 | 0.1191 | 0.1182 |
| 24h | Normoxia | | 0.2100 | 0.2453 | 0.2937 | | 0.0930 | 0.0643 | | | | 0.0617 | | 0.0899 | 0.0862 | 0.0829 |
|  | Hypoxia | | 0.3976 | 0.4250 | 0.4698 | | 0.1061 | 0.0988 | | | | 0.0973 | | 0.1413 | 0.1394 | 0.0999 |

**IRES activity (AU : LucF/LuR x 100)**

| Time | Condition | Total mean | SD | H/N ratio | M-W test  P value | Significance |
| --- | --- | --- | --- | --- | --- | --- |
| 4h | Normoxia | 13.15 | 2.23 |  |  | ns |
|  | Hypoxia | 13.57 | 2.32 | 1.03 | 0.6048 |  |
| 8h | Normoxia | 14.31 | 3.30 |  |  | ns |
|  | Hypoxia | 12.71 | 1.48 | 0.89 | 0.2973 |  |
| 24h | Normoxia | 13.63 | 8.82 |  |  | ns |
|  | Hypoxia | 21.95 | 16.04 | **1.61** | 0.0503 |  |

**I/ EMCV IRES**

| **LucF** | |  |  |  |  |  |  |  |  |  |  |
| --- | --- | --- | --- | --- | --- | --- | --- | --- | --- | --- | --- |
|  | |  |  |  |  |  |  |  |  |  |  |
|  |  | | **Biological replicates** | | | | | | | | |
| Time | | Condition | 1 | 2 | 3 | 4 | 5 | 6 | 7 | 8 | 9 |
| 4h | | Normoxia | 1 698 754 | 1 842 473 | 1 523 420 | 1 852 625 | 1 798 265 | 1 658 985 | 1 985 625 | 1 895 032 | 1 758 320 |
|  |  | Hypoxia | 1 768 791 | 1 848 197 | 1 212 320 | 1 658 986 | 1 725 542 | 1 875 258 | 1 254 214 | 1 158 220 | 1 258 210 |
| 8h | | Normoxia | 2 453 998 | 2 217 362 | 2 178 642 | 2 547 240 | 2 580 264 | 1 985 520 | 2 652 124 | 2 758 210 | 2 650 210 |
|  |  | Hypoxia | 1 098 422 | 1 388 910 | 1 053 523 | 1 658 250 | 1 752 220 | 1 425 632 | 1 452 025 | 1 252 620 | 1 158 203 |
| 24h | | Normoxia | 2 987 530 | 3 331 188 | 3 097 981 | 2 658 568 | 2 784 240 | 2 645 280 | 2 875 620 | 2 750 215 | 2 458 982 |
|  |  | Hypoxia | 3 208 903 | 3 551 010 | 3 254 552 | 2 985 568 | 3 025 106 | 3 685 210 | 3 025 240 | 2 920 213 | 3 857 920 |
|  | |  |  |  |  |  |  |  |  |  |  |
|  | |  |  |  |  |  |  |  |  |  |  |
| **LucR** | |  |  |  |  |  |  |  |  |  |  |
|  | |  |  |  |  |  |  |  |  |  |  |
|  |  | | **Biological replicates** | | | | | | | | |
| Time | | Condition | 1 | 2 | 3 | 4 | 5 | 6 | 7 | 8 | 9 |
| 4h | | Normoxia | 1 897 642 | 1 739 558 | 1 676 425 | 1 325 225 | 1 250 265 | 1 325 052 | 2 015 210 | 1 989 570 | 1 997 212 |
|  |  | Hypoxia | 1 497 245 | 1 416 380 | 1 123 235 | 998 565 | 865 258 | 1 125 580 | 1 328 620 | 1 285 210 | 1 275 487 |
| 8h | | Normoxia | 2 453 210 | 2 270 622 | 1 987 430 | 2 025 872 | 2 125 672 | 2 110 253 | 2 251 012 | 2 325 026 | 2 452 012 |
|  |  | Hypoxia | 1 345 232 | 1 423 859 | 1 125 412 | 1 257 253 | 1 520 252 | 1 427 856 | 1 352 210 | 1 245 253 | 1 124 210 |
| 24h | | Normoxia | 2 234 123 | 2 586 791 | 3 142 423 | 1 987 562 | 2 358 268 | 2 145 214 | 2 454 210 | 2 325 620 | 2 332 210 |
|  |  | Hypoxia | 2 076 764 | 1 593 918 | 2 090 845 | 2 125 147 | 2 235 126 | 1 989 987 | 2 452 012 | 2 145 217 | 2 052 982 |

| **LucF/LucR** | |  | | | |  | | |  |  |  | |  |  |  |  |
| --- | --- | --- | --- | --- | --- | --- | --- | --- | --- | --- | --- | --- | --- | --- | --- | --- |
|  |  | | **Biological replicates** | | | | | | | | | | | | | |
| Time | Condition | | 1 | 2 | 3 | | 4 | 5 | | | | 6 | | 7 | 8 | 9 |
| 4h | Normoxia | | 0.8952 | 1.0592 | 0.9087 | | 1.3980 | 1.4383 | | | | 1.2520 | | 0.9853 | 0.9525 | 0.8804 |
|  | Hypoxia | | 1.1814 | 1.3049 | 1.0793 | | 1.6614 | 1.9943 | | | | 1.6660 | | 0.9440 | 0.9012 | 0.9865 |
| 8h | Normoxia | | 1.0003 | 0.9765 | 1.0962 | | 1.2574 | 1.2139 | | | | 0.9409 | | 1.1782 | 1.1863 | 1.0808 |
|  | Hypoxia | | 0.8165 | 0.9755 | 0.9361 | | 1.3189 | 1.1526 | | | | 0.9984 | | 1.0738 | 1.0059 | 1.0302 |
| 24h | Normoxia | | 1.3372 | 1.2878 | 0.9859 | | 1.3376 | 1.1806 | | | | 1.2331 | | 1.1717 | 1.1826 | 1.0544 |
|  | Hypoxia | | 1.5451 | 2.2278 | 1.5566 | | 1.4049 | 1.3534 | | | | 1.8519 | | 1.2338 | 1.3613 | 1.8792 |

**IRES activity (AU : LucF/LuR x 100)**

| Time | Condition | Total mean | SD | H/N ratio | M-W test  P value | Significance |
| --- | --- | --- | --- | --- | --- | --- |
| 4h | Normoxia | 108.55 | 22.02 |  | 0.1903 | ns |
|  | Hypoxia | 130.21 | 38.62 | **1.20** |  |  |
| 8h | Normoxia | 110.34 | 11.29 |  | 0.2224 | ns |
|  | Hypoxia | 103.42 | 14.11 | 0.94 |  |  |
| 24h | Normoxia | 119.68 | 11.99 |  | 0.0003 | *** |
|  | Hypoxia | 160.16 | 32.22 | **1.34** |  |  |

**J/ Control without IRES (hairpin)**

**LucF**

|  |  | | Biological replicates | | | | | | | |  |  |
| --- | --- | --- | --- | --- | --- | --- | --- | --- | --- | --- | --- | --- |
| Time | Condition | 1 | 2 | 3 | 4 | 5 | 6 | 7 | 8 | 9 |  |  |
| 8 h | Normoxia | 2082 | 2490 | 1901 | 4203 | 4273 | 3176 | 5188 | 5900 | 6041 |  |  |
|  | Hypoxia | 1937 | 3592 | 2125 | 5664 | 1600 | 4811 | 3270 | 4499 | ND |  |  |
| 24 h | Normoxia | 2262 | 1232 | 1692 |  |  |  |  |  |  |  |  |
|  | Hypoxia | 2063 | 3082 | 4574 |  |  |  |  |  |  |  |  |

**LucR**

|  |  | Biological replicates | | | | | | | | | |  |  |
| --- | --- | --- | --- | --- | --- | --- | --- | --- | --- | --- | --- | --- | --- |
| Time | Condition | 1 | 2 | 3 | 4 | 5 | 6 | 7 | 8 | 9 |  |  |  |
| 8 h | Normoxia | 91411 | 99067 | 79850 | 155803 | 142912 | 127454 | 163996 | 161011 | 198604 |  |  |  |
|  | Hypoxia | 98850 | 86360 | 51747 | 100895 | 73120 | 120118 | 69871 | 75614 | ND |  |  |  |
| 24 h | Normoxia | 95815 | 50314 | 72697 |  |  |  |  |  |  |  |  |  |
|  | Hypoxia | 49263 | 68572 | 104606 |  |  |  |  |  |  |  |  |  |

| **LucF/ LucR** | | |  | |  | |  | |  |  | |  | |  | |  |
| --- | --- | --- | --- | --- | --- | --- | --- | --- | --- | --- | --- | --- | --- | --- | --- | --- |
|  |  | Biological replicates | | | | | | | | | | | |  |  |  |
| Time | Condition | 1 | | 2 | 3 | 4 | | 5 | 6 | 7 | 8 | | 9 |  |  |  |
| 8 h | Normoxia | 0.0228 | | 0.0251 | 0.0238 | 0.0270 | | 0.0299 | 0.0249 | 0.0239 | 0.0239 | | 0.0239 |  |  |  |
|  | Hypoxia | 0.0236 | | 0.0245 | 0.0233 | 0.0561 | | 0.0219 | 0.0400 | 0.0238 | 0.0238 | | 0.0238 |  |  |  |
| 24 h | Normoxia | 0.0196 | | 0.0416 | 0.0411 |  | |  |  |  |  | |  |  |  |  |
|  | Hypoxia | 0.0419 | | 0.0449 | 0.0437 |  | |  |  |  |  | |  |  |  |  |

|  |  | **AU : LucF/LucR *100** | |  |  |
| --- | --- | --- | --- | --- | --- |
| Time | Condition | Total mean (n=9) | SD | M-W test  P value | Significance |
| 8h | Normoxia | 2.80 | 0.45 | 0.4129 | ns |
|  | Hypoxia | 3.88 | 1.47 |  |  |
