# Supplementary material for: Vasohibin1, a new mouse cardiomyocyte IRES trans-acting factor that regulates translation in early hypoxia
Source: eLife. 2019 Dec 9;8:e50094. doi: 10.7554/eLife.50094 (PMC6946400; doi:10.7554/eLife.50094)
Supplement: Supplementary file 4. — (A–C) Total cell extracts from normoxic or hypoxic HL-1 cardiomyocytes were injected into the BIAcore T200 optical biosensor device where biotinylated IRES RNAs had been immobilized. The list of bound proteins identified by mass spectrometry (LC-MS/MS) after tryptic digestion is shown for FGF1 (A), VEGF-Aa (B) or EMCV (C) IRESs, respectively. The score and the number of spectra and peptides identified are indicated. For each duration of hypoxia, cells were cultivated for the same period in normoxia as a control (normoxia 4 hr and 8 hr). [file elife-50094-supp4.docx]

**Hantelys, Supplementary File 4**

**A. Proteins bound to FGF1 IRES**

**Normoxia (0 h)**

**Symbol Full name Score Spectres Peptides**

**
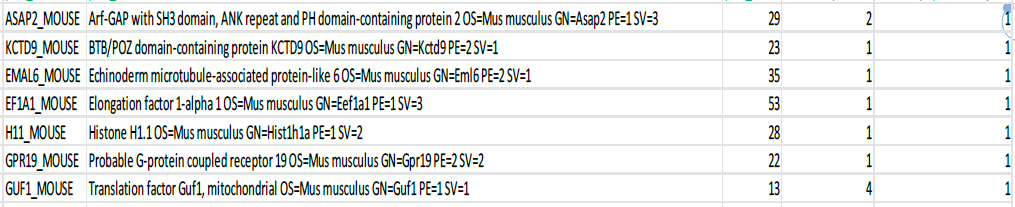
**

**Hypoxia (4 h)**

**Symbol Full name Score Spectres Peptides**

**
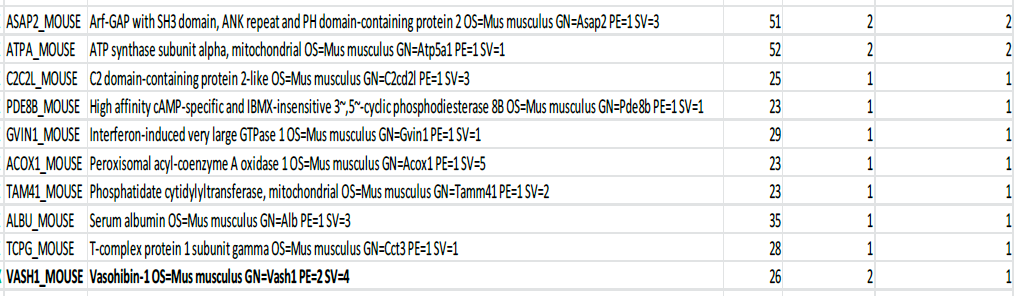
**

**Normoxia (4 h)**

**Symbol Full name Score Spectres Peptides**

**
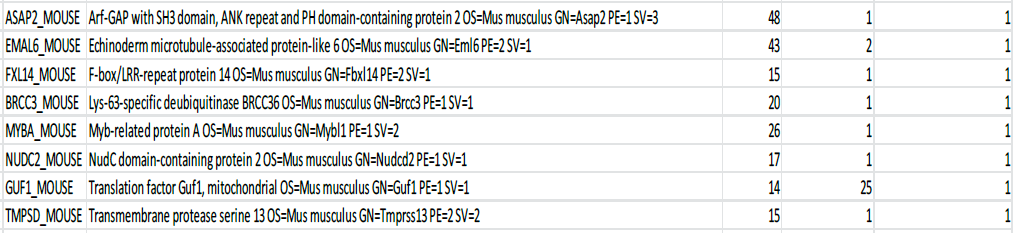
**

**Hypoxia (8 h)**

**Symbol Full name Score Spectres Peptides**

**
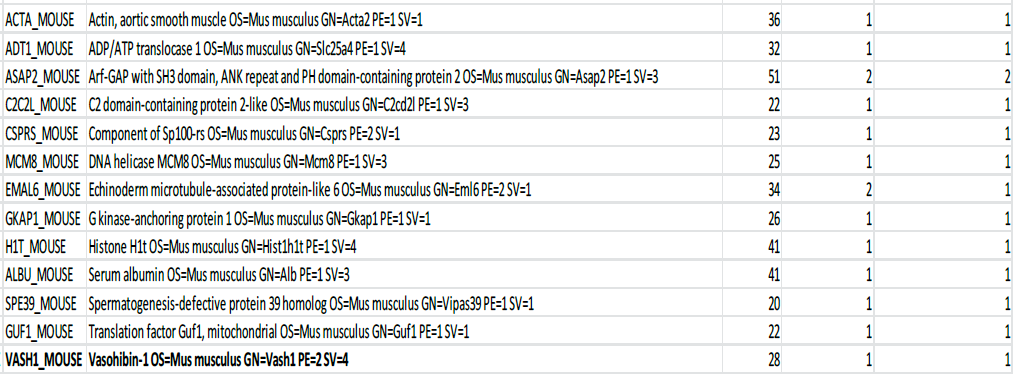
**

**Normoxia (8 h)**

**Symbol Full name Score Spectres Peptides**

**
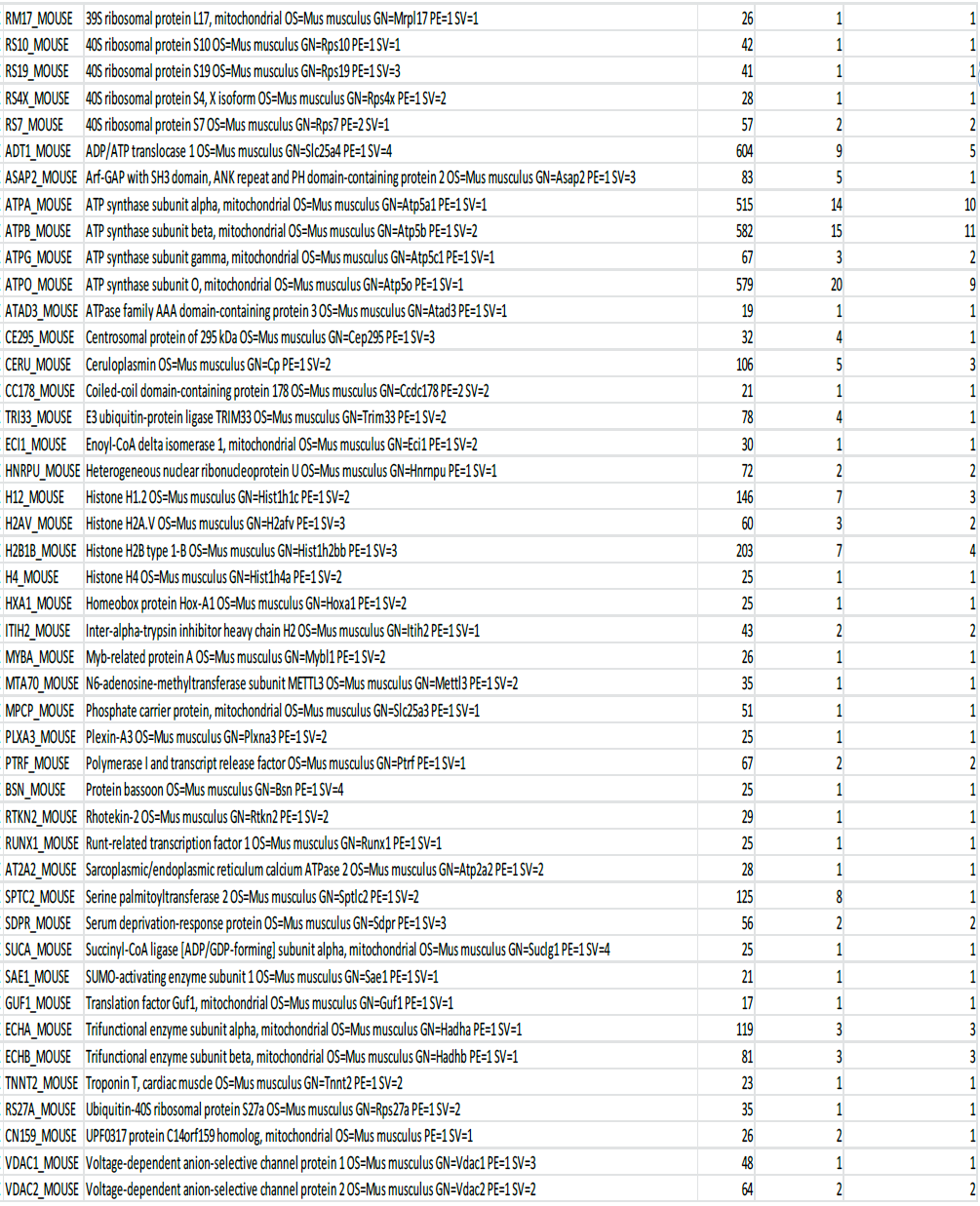
**

**B. Proteins bound to VEGFA IRES**

**Normoxia (0 h)**

**Symbol Full name Score Spectres Peptides**

**
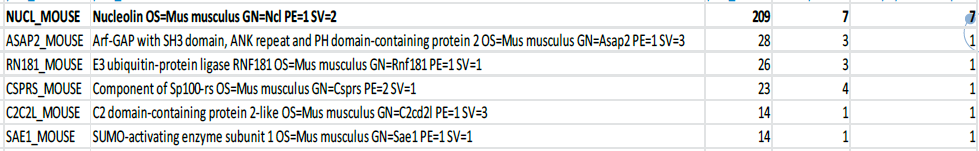
**

**Hypoxia (4 h)**

**Symbol Full name Score Spectres Peptides**

**
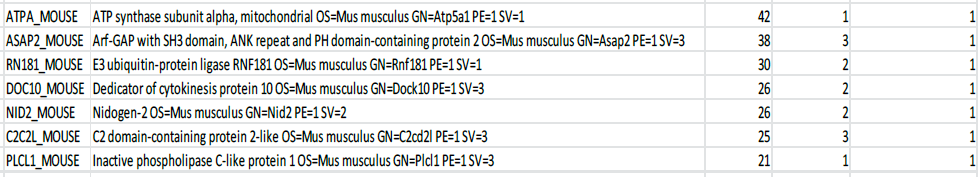
**

**Normoxia (4 h)**

**Symbol Full name Score Spectres Peptides**

**
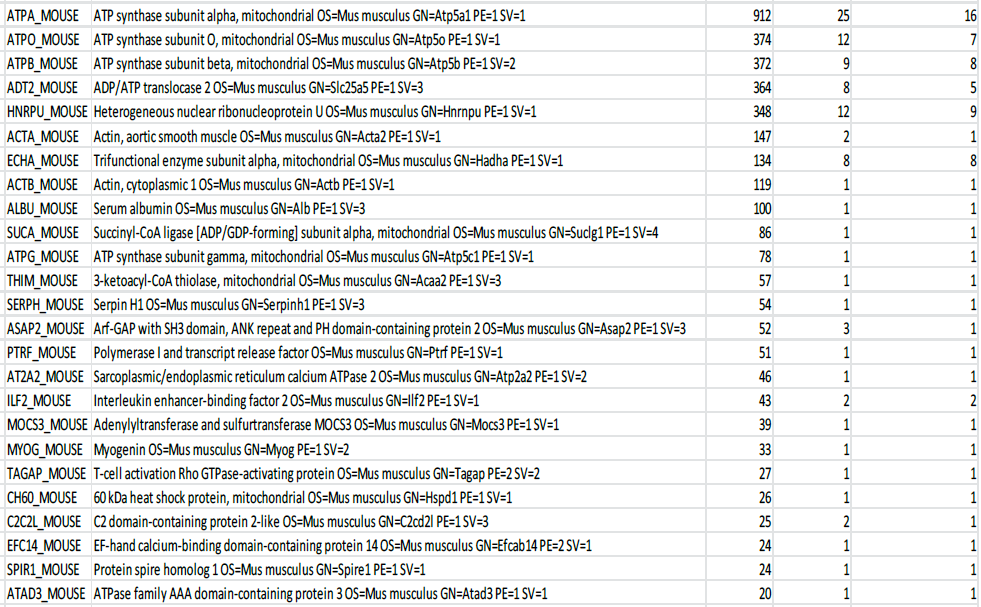
**

**Hypoxia (8 h)**

**Symbol Full name Score Spectres Peptides**

**
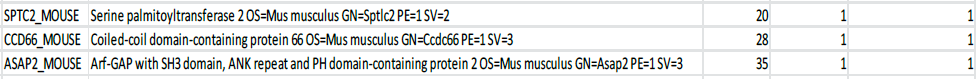
**

**Normoxia (8 h)**

**Symbol Full name Score Spectres Peptides**

**
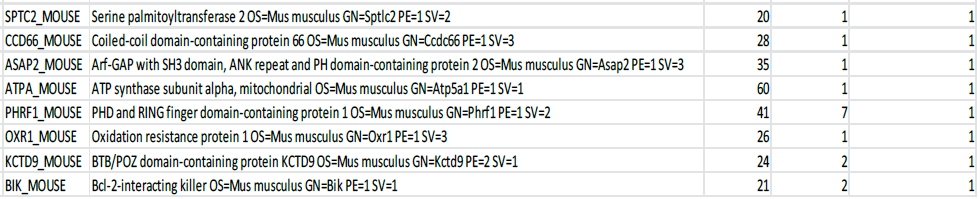
**

**C. Proteins bound to EMCV IRES**

**Normoxia (0 h)**

**Symbol Full name Score Spectres Peptides**

**
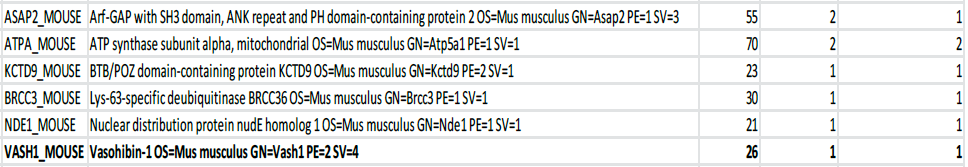
**

**Hypoxia (4 h)**

**Symbol Full name Score Spectres Peptides**

**
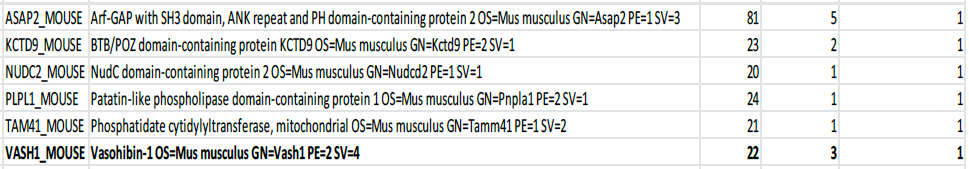
**

**Normoxia (4 h)**

**Symbol Full name Score Spectres Peptides**

**
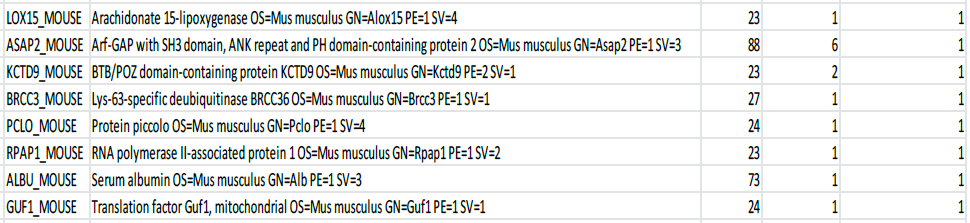
**

**Hypoxia (8 h)**

**Symbol Full name Score Spectres Peptides**

**
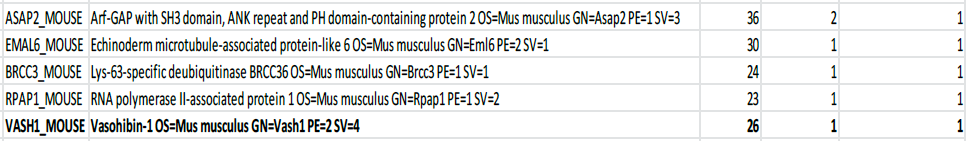
**

**Normoxia (8 h)**

**Symbol Full name Score Spectres Peptides**

**
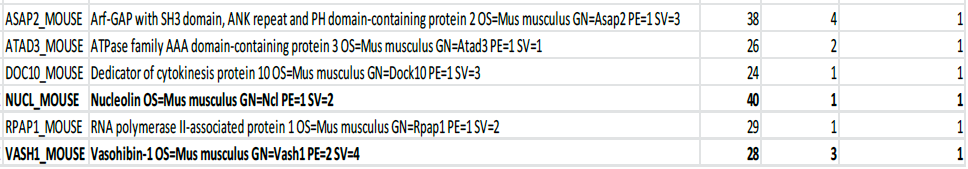
**
