# Supplementary material for: Vasohibin1, a new mouse cardiomyocyte IRES trans-acting factor that regulates translation in early hypoxia
Source: eLife. 2019 Dec 9;8:e50094. doi: 10.7554/eLife.50094 (PMC6946400; doi:10.7554/eLife.50094)
Supplement: Supplementary file 5. — HL-1 cells transduced by the different IRES-containing lentivectors were transfected with siRNA SiVASH of SiControl and submitted to 8 hr of hypoxia. Luciferase activity and IRES activities (ratio LucF/LucR x 100) were measured. For each IRES, nine biological replicates were performed with SiVASH1 or SiControl (n = 9). Each biological replicate corresponds to the mean of three technical replicates. Means, standard deviations (SD) and Mann-Whitney P values comparing IRES activities with SiVASH1 or SiControl were calculated. IRES activities corresponding to the means of all biological replicates are reported in the histograms shown in Figure 7. P-value significance is indicated: *p<0.05, **p<0.01, ns = non-significant. [file elife-50094-supp5.docx]

**Hantelys, Supplementary File 5**

**FGF1 IRES**

| **LucF** | | **Biological replicates** | | | | | | | | |
| --- | --- | --- | --- | --- | --- | --- | --- | --- | --- | --- |
|  | SiRNA | 1 | 2 | 3 | 4 | 5 | 6 | 7 | 8 | 9 |
| Normoxia | Si Control | 6165 | 5235 | 4839 | 9056 | 8546 | 8165 | 24718 | 28513 | 71475 |
|  | Si VASH1 | 6061 | 4321 | 6130 | 2819 | 4382 | 4771 | 25587 | 41809 | 41011 |
| Hypoxia | Si Control | 23268 | 23282 | ND | 9056 | 7434 | 4327 | 35934 | 27425 | 41617 |
|  | Si VASH1 | 3476 | 3798 | 3703 | 918 | 794 | 159 | 35078 | 27053 | 26439 |

| **LucR** | | **Biological replicates** | | | | | | | | |
| --- | --- | --- | --- | --- | --- | --- | --- | --- | --- | --- |
|  | SiRNA | 1 | 2 | 3 | 4 | 5 | 6 | 7 | 8 | 9 |
| Normoxia | Si Control | 31326 | 43193 | 26638 | 18202 | 41546 | 215819 | 361595 | 537143 | 71475 |
|  | Si VASH1 | 25524 | 46369 | 20966 | 30708 | 37007 | 169661 | 310210 | 232371 | 41011 |
| Hypoxia | Si Control | 28719 | 24302 | 47957 | 40352 | 28489 | 166883 | 126093 | 212489 | 41617 |
|  | Si VASH1 | 14606 | 17491 | 6722 | 7810 | 5492 | 202073 | 157173 | 139745 | 26439 |

| **LucF/LucR** | | **Biological replicates** | | | | | | | | |
| --- | --- | --- | --- | --- | --- | --- | --- | --- | --- | --- |
|  | SiRNA | 1 | 2 | 3 | 4 | 5 | 6 | 7 | 8 | 9 |
| Normoxia | Si Control | 0.1154 | 0.1671 | 0.1120 | 0.1325 | 0.1124 | 0.1632 | 0.1145 | 0.0789 | 0.1331 |
|  | Si VASH1 | 0.1235 | 0.1379 | 0.1419 | 0.0954 | 0.1482 | 0.1614 | 0.1508 | 0.1348 | 0.1765 |
| Hypoxia | Si Control | 1.1574 | 0.8107 | ND | 0.1888 | 0.1840 | 0.1520 | 0.2153 | 0.2175 | 0.1959 |
|  | Si VASH1 | 0.1729 | 0.1323 | 0.1524 | 0.1364 | 0.1016 | 0.0289 | 0.1736 | 0.1721 | 0.1892 |

| \| **IRES activities (AU : LucF/LucR x 100)** \| \| \| \| \| **Normalized to Si control** \| \| \|  \|  \| \| --- \| --- \| --- \| --- \| --- \| --- \| --- \| --- \| --- \| --- \| \| Time \| Condition \| Total mean \| SD \| Ratio \| SD \| M-W P value \| Significance \| \| Normoxia \| Si Control \| 12.54 \| 2.74 \| 1.00 \| 0.22 \|  \|  \| \| Si VASH1 \| 14.12 \| 2.31 \| **1.13** \| **0.18** \| **0.2973** \| ns \| \| Hypoxia \| Si Control \| 39.02 \| 37.86 \| 1.00 \| 0.97 \|  \|  \| \| Si VASH1 \| 13.99 \| 4.96 \| **0.36** \| **0.13** \| **0.0037** \| ** \| |
| --- | --- | --- | --- | --- | --- | --- | --- | --- | --- | --- | --- | --- | --- | --- | --- | --- | --- | --- | --- | --- | --- | --- | --- | --- | --- | --- | --- | --- | --- | --- | --- | --- | --- | --- | --- | --- | --- | --- | --- | --- | --- | --- | --- | --- | --- | --- | --- | --- |
|  |

|  |
| --- |

**FGF2 IRES**

| **LucF** | | **Biological replicates** | | | | | | | | |
| --- | --- | --- | --- | --- | --- | --- | --- | --- | --- | --- |
|  | SiRNA | 1 | 2 | 3 | 4 | 5 | 6 | 7 | 8 | 9 |
| Normoxia | Si Control | 22826 | 21218 | 12844 | 1378436 | 1082836 | 1164807 | 83190 | 93828 | 99601 |
|  | Si VASH1 | 21094 | 24819 | 20555 | 2209438 | 826675 | 1840332 | 67392 | 76305 | 73470 |
| Hypoxia | Si Control | 19965 | 12844 | 11697 | 1017433 | 1179299 | 829663 | 67103 | 78868 | 71632 |
|  | Si VASH1 | 15522 | 15043 | 16528 | 893314 | 749103 | 966069 | 59989 | 68906 | 68663 |

| **LucR** | | **Biological replicates** | | | | | | | | |
| --- | --- | --- | --- | --- | --- | --- | --- | --- | --- | --- |
|  | SiRNA | 1 | 2 | 3 | 4 | 5 | 6 | 7 | 8 | 9 |
| Normoxia | Si Control | 180511 | 135180 | 129077 | 12202038 | 11842478 | 11986298 | 590388 | 751723 | 757328 |
|  | Si VASH1 | 125421 | 125058 | 135793 | 14135403 | 8953356 | 10851103 | 556354 | 613876 | 606021 |
| Hypoxia | Si Control | 96016 | 67708 | 75468 | 6222023 | 7669135 | 5269169 | 334572 | 394644 | 375540 |
|  | Si VASH1 | 68476 | 61198 | 64605 | 4535768 | 4011896 | 5170671 | 392981 | 376414 | 347094 |

| **LucF/LucR** | | **Biological replicates** | | | | | | | | |
| --- | --- | --- | --- | --- | --- | --- | --- | --- | --- | --- |
|  | SiRNA | 1 | 2 | 3 | 4 | 5 | 6 | 7 | 8 | 9 |
| Normoxia | Si Control | 0.1265 | 0.1570 | 0.0995 | 0.1130 | 0.0914 | 0.0972 | 0.1409 | 0.1248 | 0.1315 |
|  | Si VASH1 | 0.1169 | 0.1836 | 0.1592 | 0.1563 | 0.0923 | 0.1696 | 0.1211 | 0.1243 | 0.1212 |
| Hypoxia | Si Control | 0.2079 | 0.1897 | 0.1550 | 0.1635 | 0.1538 | 0.1575 | 0.2006 | 0.1998 | 0.1907 |
|  | Si VASH1 | 0.1617 | 0.2222 | 0.2190 | 0.1969 | 0.1867 | 0.1868 | 0.1526 | 0.1831 | 0.1978 |

| **IRES activities (AU : LucF/LucR x 100)** | | | | **Normalized to Si control** | | |  | |  |
| --- | --- | --- | --- | --- | --- | --- | --- | --- | --- |
| Time | Condition | Total mean | SD | Ratio | SD | M-W P value | Significance |  |  |
| Normoxia | Si Control | 12.02 | 2.18 | 1.00 | 0.18 |  |  |  |  |
|  | Si VASH1 | 13.83 | 2.99 | **1.15** | **0.25** | **0.3401** | ns |  |  |
| Hypoxia | Si Control | 17.98 | 2.21 | 1.00 | 0.12 |  |  |  |  |
|  | Si VASH1 | 18.97 | 2.31 | **1.05** | **0.13** | **0.7304** | ns |  |  |

**VEGFA IRES a**

| **LucF** | | **Biological replicates** | | | | | | | | |
| --- | --- | --- | --- | --- | --- | --- | --- | --- | --- | --- |
|  | SiRNA | 1 | 2 | 3 | 4 | 5 | 6 | 7 | 8 | 9 |
| Normoxia | Si Control | 1063 | 768 | 835 | 10177 | 10903 | 9795 | 8927 | 10090 | 10353 |
|  | Si VASH1 | ND | ND | ND | 12008 | 10355 | 13047 | 7081 | 6763 | 7085 |
| Hypoxia | Si Control | 944 | 938 | 958 | 21334 | 25229 | 24602 | 6919 | 8893 | 10550 |
|  | Si VASH1 | 985 | 1284 | 1040 | 18934 | 25104 | 26388 | 6087 | 5714 | 5375 |

| **LucR** | | **Biological replicates** | | | | | | | | | |
| --- | --- | --- | --- | --- | --- | --- | --- | --- | --- | --- | --- |
|  | SiRNA | 1 | 2 | 3 | 4 | 5 | 6 | 7 | 8 | 9 |  |
| Normoxia | Si Control | 110313 | 88187 | 88187 | 858315 | 1006460 | 1131780 | 800801 | 851785 | 976533 |  |
|  | Si VASH1 | 104087 | 104234 | 88328 | 1122644 | 949786 | 1245834 | 781558 | 904997 | 1041730 |  |
| Hypoxia | Si Control | 110313 | 88187 | 88187 | 1201671 | 1239986 | 1269291 | 397755 | 412761 | 462290 |  |
|  | Si VASH1 | 104087 | 104234 | 88328 | 1178924 | 1314113 | 1411139 | 397766 | 338569 | 370795 |  |

| **LucF/LucR** | | **Biological replicates** | | | | | | | | |
| --- | --- | --- | --- | --- | --- | --- | --- | --- | --- | --- |
|  | SiRNA | 1 | 2 | 3 | 4 | 5 | 6 | 7 | 8 | 9 |
| Normoxia | Si Control | 0.0084 | 0.0083 | 0.0091 | 0.0119 | 0.0108 | 0.0087 | 0.0111 | 0.0118 | 0.0106 |
|  | Si VASH1 | ND | ND | ND | 0.0107 | 0.0109 | 0.0105 | 0.0089 | 0.0098 | 0.0101 |
| Hypoxia | Si Control | 0.0086 | 0.0106 | 0.0109 | 0.0178 | 0.0203 | 0.0194 | 0.0178 | 0.0164 | 0.0153 |
|  | Si VASH1 | 0.0089 | 0.0146 | 0.0118 | 0.0161 | 0.0191 | 0.0187 | 0.0153 | 0.0169 | 0.0145 |

| **IRES activities (AU : LucF/LucR x 100)** | | | | **Normalized to Si control** | | |  | |  |
| --- | --- | --- | --- | --- | --- | --- | --- | --- | --- |
| Time | Condition | Total mean | SD | Ratio | SD | M-W P value | Significance |  |  |
| Normoxia | Si Control | 1.01 | 0.15 | 1.00 | 0.14 |  |  |  |  |
|  | Si VASH1 | 1.01 | 0.07 | **1.01** | **0.07** | **0.9546** | ns |  |  |
| Hypoxia | Si Control | 1.52 | 0.42 | 1.00 | 0.28 |  |  |  |  |
|  | Si VASH1 | 1.51 | 0.32 | **0.99** | **0.21** | **0.7464** | ns |  |  |

**VEGFA IRES b**

| **LucF** | | **Biological replicates** | | | | | | | | |
| --- | --- | --- | --- | --- | --- | --- | --- | --- | --- | --- |
|  | SiRNA | 1 | 2 | 3 | 4 | 5 | 6 | 7 | 8 | 9 |
| Normoxia | Si Control | 24407 | 29293 | 28866 | 636888 | 673362 | 711456 | 336029 | 485230 | 456130 |
|  | Si VASH1 | 26057 | 23895 | 24286 | 754530 | 811206 | 796490 | 377720 | 490184 | 368048 |
| Hypoxia | Si Control | 25539 | 27250 | 27360 | 1455967 | 1495875 | 1461388 | 252396 | 325862 | 292610 |
|  | Si VASH1 | 29633 | 27197 | 27734 | 1442573 | 1496275 | 1406109 | 290051 | 301154 | 292703 |

| **LucR** | | **Biological replicates** | | | | | | | | |
| --- | --- | --- | --- | --- | --- | --- | --- | --- | --- | --- |
|  | SiRNA | 1 | 2 | 3 | 4 | 5 | 6 | 7 | 8 | 9 |
| Normoxia | Si Control | 204606 | 259243 | 265657 | 3953413 | 4461082 | 4955118 | 2785870 | 3180830 | 2979085 |
|  | Si VASH1 | 129783 | 131937 | 114093 | 4588230 | 4469611 | 4706020 | 3035339 | 3373906 | 2706872 |
| Hypoxia | Si Control | 207937 | 216079 | 211792 | 5227968 | 5137726 | 5358864 | 1206699 | 1464757 | 1496579 |
|  | Si VASH1 | 179775 | 171019 | 145280 | 5346747 | 5752000 | 5164154 | 1221397 | 1296195 | 1237799 |

| **LucF/LucR** | | **Biological replicates** | | | | | | | | |
| --- | --- | --- | --- | --- | --- | --- | --- | --- | --- | --- |
|  | SiRNA | 1 | 2 | 3 | 4 | 5 | 6 | 7 | 8 | 9 |
| Normoxia | Si Control | 0.1193 | 0.1130 | 0.1087 | 0.1611 | 0.1509 | 0.1436 | 0.1206 | 0.1525 | 0.1531 |
|  | Si VASH1 | 0.1274 | 0.0922 | 0.0914 | 0.1644 | 0.1815 | 0.1692 | 0.1244 | 0.1453 | 0.1360 |
| Hypoxia | Si Control | 0.1228 | 0.1261 | 0.1292 | 0.2785 | 0.2912 | 0.2727 | 0.2092 | 0.2225 | 0.1955 |
|  | Si VASH1 | 0.1425 | 0.1259 | 0.1309 | 0.2698 | 0.2601 | 0.2723 | 0.2375 | 0.2323 | 0.2365 |

| **IRES activities (AU : LucF/LucR x 100)** | | | | **Normalized to Si control** | | |  | |  |
| --- | --- | --- | --- | --- | --- | --- | --- | --- | --- |
| Time | Condition | Total mean | SD | Ratio | SD | M-W P value | Significance |  |  |
| Normoxia | Si Control | 13.59 | 2.02 | 1.00 | 0.15 |  |  |  |  |
|  | Si VASH1 | 13.69 | 3.20 | **1.01** | **0.24** | **0.7962** | ns |  |  |
| Hypoxia | Si Control | 20.53 | 6.76 | 1.00 | 0.33 |  |  |  |  |
|  | Si VASH1 | 21.20 | 6.10 | **1.03** | **0.30** | **0.8633** | ns |  |  |

**VEGFC IRES**

| **LucF** | | **Biological replicates** | | | | | | | | |
| --- | --- | --- | --- | --- | --- | --- | --- | --- | --- | --- |
|  | SiRNA | 1 | 2 | 3 | 4 | 5 | 6 | 7 | 8 | 9 |
| Normoxia | Si Control | 246375 | 242933 | 314902 | 217056 | 137257 | 253247 | 34901 | 43369 | 30196 |
|  | Si VASH1 | 487878 | 477149 | 492814 | 229290 | 246978 | 182056 | 38245 | 41693 | 27884 |
| Hypoxia | Si Control | 335045 | 434617 | 425637 | 281808 | 313489 | 279125 | 24456 | 25053 | 23295 |
|  | Si VASH1 | 356286 | 400146 | 398669 | 307224 | 258394 | 357857 | 20099 | 20436 | 22661 |

| **LucR** | | **Biological replicates** | | | | | | | | |
| --- | --- | --- | --- | --- | --- | --- | --- | --- | --- | --- |
|  | SiRNA | 1 | 2 | 3 | 4 | 5 | 6 | 7 | 8 | 9 |
| Normoxia | Si Control | 3487442 | 3595724 | 2160847 | 1940200 | 1317594 | 1986534 | 517859 | 522417 | 438634 |
|  | Si VASH1 | 5663195 | 5510976 | 2230869 | 1895114 | 2069968 | 1309904 | 562597 | 580376 | 487313 |
| Hypoxia | Si Control | 3743871 | 4296534 | 1914906 | 2035704 | 2389202 | 2247759 | 288505 | 254400 | 261953 |
|  | Si VASH1 | 3382601 | 3988748 | 1245215 | 2242942 | 2337124 | 2558068 | 190852 | 185250 | 218449 |

| **LucF/LucR** | | **Biological replicates** | | | | | | | | |
| --- | --- | --- | --- | --- | --- | --- | --- | --- | --- | --- |
|  | SiRNA | 1 | 2 | 3 | 4 | 5 | 6 | 7 | 8 | 9 |
| Normoxia | Si Control | 0.0706 | 0.0676 | 0.1457 | 0.1119 | 0.1042 | 0.1275 | 0.0674 | 0.0830 | 0.0688 |
|  | Si VASH1 | 0.0861 | 0.0866 | 0.2209 | 0.1210 | 0.1193 | 0.1390 | 0.0680 | 0.0718 | 0.0572 |
| Hypoxia | Si Control | 0.0895 | 0.1012 | 0.2223 | 0.1384 | 0.1312 | 0.1242 | 0.0848 | 0.0985 | 0.0889 |
|  | Si VASH1 | 0.1053 | 0.1003 | 0.3202 | 0.1370 | 0.1106 | 0.1399 | 0.1053 | 0.1103 | 0.1037 |

| **IRES activities (AU : LucF/LucR x 100)** | | | | **Normalized to Si control** | | |  | |  |
| --- | --- | --- | --- | --- | --- | --- | --- | --- | --- |
| Time | Condition | Total mean | SD | Ratio | SD | M-W P value | Significance |  |  |
| Normoxia | Si Control | 9.41 | 2.94 | 1.00 | 0.31 |  |  |  |  |
|  | Si VASH1 | 10.78 | 5.05 | **1.15** | **0.54** | **0.6048** | ns |  |  |
| Hypoxia | Si Control | 11.99 | 4.32 | 1.00 | 0.36 |  |  |  |  |
|  | Si VASH1 | 13.70 | 7.02 | **1.14** | **0.59** | **0.2867** | ns |  |  |

**VEGFD IRES**

| **LucF** | | **Biological replicates** | | | | | | | | |
| --- | --- | --- | --- | --- | --- | --- | --- | --- | --- | --- |
|  | SiRNA | 1 | 2 | 3 | 4 | 5 | 6 | 7 | 8 | 9 |
| Normoxia | Si Control | 145323 | 155346 | 154191 | 47796 | 48288 | 42436 | 75842 | 68756 | 63160 |
|  | Si VASH1 | 194207 | 225219 | 224077 | 41472 | 53177 | 48922 | 50506 | 69895 | 67848 |
| Hypoxia | Si Control | 195382 | 241996 | 212936 | 86899 | 103834 | 88966 | 38323 | 41266 | 45431 |
|  | Si VASH1 | 179053 | 197152 | 112369 | 79555 | 81056 | 77725 | 29441 | 36166 | 41950 |

| **LucR** | | **Biological replicates** | | | | | | | | |
| --- | --- | --- | --- | --- | --- | --- | --- | --- | --- | --- |
|  | SiRNA | 1 | 2 | 3 | 4 | 5 | 6 | 7 | 8 | 9 |
| Normoxia | Si Control | 1907458 | 2093765 | 2160847 | 567674 | 597668 | 541654 | 858060 | 837030 | 638997 |
|  | Si VASH1 | 1991236 | 2298870 | 2230869 | 517993 | 560985 | 512755 | 593777 | 780959 | 789452 |
| Hypoxia | Si Control | 1619167 | 1897011 | 1914906 | 525002 | 615275 | 500416 | 326465 | 350347 | 383692 |
|  | Si VASH1 | 1641814 | 1672889 | 1245215 | 536248 | 545353 | 584930 | 278668 | 318382 | 374719 |

| **LucF/LucR** | | **Biological replicates** | | | | | | | | |
| --- | --- | --- | --- | --- | --- | --- | --- | --- | --- | --- |
|  | SiRNA | 1 | 2 | 3 | 4 | 5 | 6 | 7 | 8 | 9 |
| Normoxia | Si Control | 0.0762 | 0.0742 | 0.0714 | 0.0842 | 0.0808 | 0.0783 | 0.0884 | 0.0821 | 0.0988 |
|  | Si VASH1 | 0.0975 | 0.0980 | 0.1004 | 0.0801 | 0.0948 | 0.0954 | 0.0851 | 0.0895 | 0.0859 |
| Hypoxia | Si Control | 0.1207 | 0.1276 | 0.1112 | 0.1655 | 0.1688 | 0.1778 | 0.1174 | 0.1178 | 0.1184 |
|  | Si VASH1 | 0.1091 | 0.1179 | 0.0902 | 0.1484 | 0.1486 | 0.1329 | 0.1057 | 0.1136 | 0.1120 |

| **IRES activities (AU : LucF/LucR x 100)** | | | | **Normalized to Si control** | | | |  |  |
| --- | --- | --- | --- | --- | --- | --- | --- | --- | --- |
| Time | Condition | Total mean | SD | Ratio | SD | M-W P value | Significance |  |  |
| Normoxia | Si Control | 8.16 | 0.83 | 1.00 | 0.10 |  |  |  |  |
|  | Si VASH1 | 9.19 | 0.70 | **1.13** | **0.09** | **0.0188** | * |  |  |
| Hypoxia | Si Control | 13.61 | 2.65 | 1.00 | 0.19 |  |  |  |  |
|  | Si VASH1 | 11.98 | 1.97 | **0.88** | **0.14** | **0.1359** | ns |  |  |

**c-myc IRES**

| **LucF** | | **Biological replicates** | | | | | | | | |
| --- | --- | --- | --- | --- | --- | --- | --- | --- | --- | --- |
|  | SiRNA | 1 | 2 | 3 | 4 | 5 | 6 | 7 | 8 | 9 |
| Normoxia | Si Control | 252109 | 291920 | 918687 | 325307 | 384619 | 326642 | 249643 | 233046 | 183453 |
|  | Si VASH1 | 1424225 | 1180035 | 1295701 | 352401 | 300184 | 317795 | 186267 | 182128 | 179026 |
| Hypoxia | Si Control | 816497 | 422387 | 80789 | 575691 | 601917 | 534370 | 183838 | 194270 | 220011 |
|  | Si VASH1 | 622268 | 530019 | 497331 | 518194 | 406193 | 591820 | 178636 | 177011 | 163963 |

| **LucR** | | **Biological replicates** | | | | | | | | |
| --- | --- | --- | --- | --- | --- | --- | --- | --- | --- | --- |
|  | SiRNA | 1 | 2 | 3 | 4 | 5 | 6 | 7 | 8 | 9 |
| Normoxia | Si Control | 2603194 | 2581366 | 4956962 | 1216742 | 1371688 | 1207546 | 1738743 | 1532354 | 1438325 |
|  | Si VASH1 | 6466612 | 5522384 | 5429788 | 1197183 | 1080280 | 1156255 | 1513541 | 1375704 | 1410980 |
| Hypoxia | Si Control | 3585096 | 2670578 | 530645 | 1405500 | 1480296 | 1467212 | 775271 | 746257 | 985294 |
|  | Si VASH1 | 2552808 | 2119514 | 2073981 | 1449064 | 1365072 | 1405441 | 868202 | 777132 | 863443 |

| **LucF/LucR** | | **Biological replicates** | | | | | | | | |
| --- | --- | --- | --- | --- | --- | --- | --- | --- | --- | --- |
|  | SiRNA | 1 | 2 | 3 | 4 | 5 | 6 | 7 | 8 | 9 |
| Normoxia | Si Control | 0.0968 | 0.1131 | 0.1853 | 0.2674 | 0.2804 | 0.2705 | 0.1436 | 0.1521 | 0.1275 |
|  | Si VASH1 | 0.2202 | 0.2137 | 0.2386 | 0.2944 | 0.2779 | 0.2748 | 0.1231 | 0.1324 | 0.1269 |
| Hypoxia | Si Control | 0.2277 | 0.1582 | 0.1522 | 0.4096 | 0.4066 | 0.3642 | 0.2371 | 0.2603 | 0.2233 |
|  | Si VASH1 | 0.2438 | 0.2501 | 0.2398 | 0.3576 | 0.2976 | 0.4211 | 0.2058 | 0.2278 | 0.1899 |

| **IRES activities (AU : LucF/LucR x 100)** | **Normalized to Si control** |  | |  |
| --- | --- | --- | --- | --- |
| \| Time \| Condition \| Total mean \| SD \| Ratio \| SD \| M-W P value \| Significance \| \| --- \| --- \| --- \| --- \| --- \| --- \| --- \| --- \| \| Normoxia \| Si Control \| 18.19 \| 7.26 \| 1.00 \| 0.40 \|  \|  \| \| Si VASH1 \| 21.13 \| 6.83 \| **1.16** \| **0.38** \| **0.4363** \| ns \| \| Hypoxia \| Si Control \| 27.10 \| 9.91 \| 1.00 \| 0.37 \|  \|  \| \| Si VASH1 \| 27.04 \| 7.55 \| **1.00** \| **0.28** \| **0.7962** \| ns \| | | | |  |

**EMCV IRES**

| **LucF** | | **Biological replicates** | | | | | | | | |
| --- | --- | --- | --- | --- | --- | --- | --- | --- | --- | --- |
|  | SiRNA | 1 | 2 | 3 | 4 | 5 | 6 | 7 | 8 | 9 |
| Normoxia | Si Control | 1702630 | 1760136 | 1302068 | 107301 | 144501 | 143440 | 330170 | 293299 | 266000 |
|  | Si VASH1 | 5352755 | 7029602 | 3647215 | 112778 | 121482 | 113111 | 291868 | 245382 | 237669 |
| Hypoxia | Si Control | 2686430 | 2928766 | 2764654 | 223538 | 216316 | 233362 | 244519 | 254123 | 209543 |
|  | Si VASH1 | ND | 886021 | 2250249 | 221733 | 209587 | 204733 | 216546 | 204516 | 186109 |

| **LucR** | | **Biological replicates** | | | | | | | | |
| --- | --- | --- | --- | --- | --- | --- | --- | --- | --- | --- |
|  | SiRNA | 1 | 2 | 3 | 4 | 5 | 6 | 7 | 8 | 9 |
| Normoxia | Si Control | 2208003 | 2016725 | 1796654 | 147772 | 179110 | 161146 | 365365 | 327375 | 301181 |
|  | Si VASH1 | 5336122 | 6838676 | 2959448 | 144691 | 146769 | 138309 | 316197 | 262296 | 254808 |
| Hypoxia | Si Control | 2156164 | 2373157 | 1936666 | 177395 | 182885 | 198846 | 173211 | 189437 | 175377 |
|  | Si VASH1 | ND | 730239 | 1752792 | 191909 | 168829 | 202128 | 170625 | 166261 | 149249 |

| **LucF/LucR** | | **Biological replicates** | | | | | | | | |
| --- | --- | --- | --- | --- | --- | --- | --- | --- | --- | --- |
|  | SiRNA | 1 | 2 | 3 | 4 | 5 | 6 | 7 | 8 | 9 |
| Normoxia | Si Control | 0.7711 | 0.8728 | 0.7247 | 0.7261 | 0.8068 | 0.8901 | 0.9037 | 0.8959 | 0.8832 |
|  | Si VASH1 | 1.0031 | 1.0279 | 1.2324 | 0.7794 | 0.8277 | 0.8178 | 0.9231 | 0.9355 | 0.9327 |
| Hypoxia | Si Control | 1.2459 | 1.2341 | 1.4275 | 1.2601 | 1.1828 | 1.1736 | 1.4117 | 1.3415 | 1.1948 |
|  | Si VASH1 | ND | 1.2133 | 1.2838 | 1.1554 | 1.2414 | 1.0129 | 1.2691 | 1.2301 | 1.2470 |

| **IRES activities (AU : LucF/LucR x 100)** | | | | **Normalized to Si control** | | | |  |  |
| --- | --- | --- | --- | --- | --- | --- | --- | --- | --- |
| Time | Condition | Total mean | SD | Ratio | SD | M-W P value | Significance |  |  |
| Normoxia | Si Control | 83.05 | 7.41 | 1.00 | 0.09 |  |  |  |  |
|  | Si VASH1 | 94.22 | 13.73 | **1.13** | **0.17** | **0.0315** | * |  |  |
| Hypoxia | Si Control | 127.47 | 9.64 | 1.00 | 0.08 |  |  |  |  |
|  | Si VASH1 | 120.66 | 8.74 | **0.95** | **0.07** | **0.4234** | ns |  |  |
