# Supplementary material for: Vasohibin1, a new mouse cardiomyocyte IRES trans-acting factor that regulates translation in early hypoxia
Source: eLife. 2019 Dec 9;8:e50094. doi: 10.7554/eLife.50094 (PMC6946400; doi:10.7554/eLife.50094)
Supplement: Supplementary file 6. [file elife-50094-supp6.docx]

Hantelys et al, Supplementary File 6

| **Target** | **Forward primer 5' to 3'** | **Reverse primer 5' to 3'** |
| --- | --- | --- |
| *Akt1* | AGAACTCTAGGCATCCCTTCC | CGTTGGCATACTCCATGACA |
| *Ang* | TCCTGACTCAGCACCATGAC | ACATCTTTGCAGGGTGAGGTTA |
| *Angpt1* | ACAACACCGGGAAGATGGAA | TTCACCAGAGGGATTCCCAAAA |
| *Angpt2* | GAACCAGACAGCAGCACAAA | TCGAGTCTTGTCGTCTGGTTTA |
| *Angptl4* | CTTGGGACCAAGACCATGAC | TGGCTACAGGTACCAAACCA |
| *Anpep* | TGGGACTTTGTCCGAAGCA | TCCCTGGATGAGATTGGCAAA |
| *Apln (Apelin)* | GCAGGAGGAAATTTCGCAGAC | ACTTGGCGAGCCCTTCAA |
| *Aplnr* | TTGACTGGCCTTTTGGAACC | GCAAAAGACACTGGCGTACA |
| *Atp2a2* | CGGTCCAAGAGTCTCCTTCTA | GCACAATCCACTCCATCGAA |
| *Bai1* | GGTCCTGAGAAGCAAACCAA | GACCATTCGTTCCAGTTTCCA |
| *Ccl11(Eotaxin)* | CAACAACAGATGCACCCTGAA | CACAGATCTCTTTGCCCAACC |
| *Ccl2 (mcp-1)* | AGCAGCAGGTGTCCCAAA | TTCTTGGGGTCAGCACAGAC |
| *Ccl21a* | GTCAGGACTGCTGCCTTAAGTA | GCTTCCTATAGCCTCGGACAA |
| *Cdh5* | AACGAGGACAGCAACTTCAC | TGGCATGCTCCCGATTAAAC |
| *Col18a1* | CAGGACCAAAGGGTGACAAA | TTCCAGGTGGAAGAGGTCAA |
| *Col4a3* | GCTGGTACAAAGGGCAACAA | TAAGCCTGGCAATCCATCCA |
| *Ctgf* | AAGCTGACCTGGAGGAAAACA | TGCAGCCAGAAAGCTCAAAC |
| *Cxcl1* | CCTGAAGCTCCCTTGGTTCA | TTCTCCGTTACTTGGGGACAC |
| *Cxcl10 (Inp10)* | ATCCGGAATCTAAGACCATCAAGAA | GCTCTCTGCTGTCCATCCA |
| *Cxcl5 (ena78/lix)* | GGCATTTCTGTTGCTGTTCAC | TGCGGCTATGACTGAGGAA |
| *Cxcl9* | AGCCCCAATTGCAACAAAAC | TCTTCACATTTGCCGAGTCC |
| *Cyr61* | CCACACCAAGGGGTTGGAA | CACAGGGTCTGCCTTCTGAC |
| *Edn1* | CCTGGACATCATCTGGGTCAA | AACGCTTGGACCTGGAAGAA |
| *Efna1* | TGGGCAAGGAGTTCAAGGAA | GCACTGGGATTCCTGATGGTA |
| *Efnb2* | TGCCAGACAAGAGCCATGAA | GTCTTGTTGGACCGTGATTCC |
| *Egf* | GGAGAGACTGCTGAGTGTCA | AGCCAGCACACACTCATCTA |
| *Eng* | AGGCATCCAACACCATCGAA | TCTAGCTGGACTGTGACCTCA |
| *Ephb4* | CCTCACGGAATTCATGGAGAAC | ACCAGCTGGATGACTGTGAA |
| *Erbb2 (Her2)* | ATTCTCAGACGCCGGTTCA | TTGGCCCCAAAGGTCATCA |
| *F3* | ACCCAAACCCACCAACTATACC | GTGTCTGTGGTCGAGAAGCA |
| *Fgf1 (aFGF)* | TGGACACCGAAGGGCTTTTA | GCATGCTTCTTGGAGGTGTAA |
| *Fgf2 (bFGF)* | TCTTCCTGCGCATCCATCC | GCACACACTCCCTTGATAGACA |
| *Fgfr3* | AGGATTTAGACCGCATCCTCAC | CCTGGCGAGTACTGCTCAAA |
| *Flt1* | TTGCACGGGAGAGACTGAAA | GCCAAATGCAGAGGCTTGAA |
| *Fn1* | CGTCATTGCCCTGAAGAACA | AAGGGTAACCAGTTGGGGAA |
| *Hgf* | CATCAAATGCCAGCCTTGGAA | TCTTTACCGCGATAGCTCGAA |
| *Hif1a* | TCGACACAGCCTCGATATGAA | TTCCGGCTCATAACCCATCA |
| *Hnrnpm* | GATGCCAACCATCTGAGCAAA | CCAAATCCTATGCCTTCCATTCC |
| *Hpse* | GCCTCGAGGGAAGACAGTTAAA | TGCCATGTAAGAGAGTCGATCAC |
| *Id1* | ACCCTGAACGGCGAGATCA | GATCGTCGGCTGGAACACA |
| *Ifna1* | TCCACCAGCAGCTCAATGAC | TCTTCCTGGGTCAGGGGAAA |
| *Ifng* | GGCACAGTCATTGAAAGCCTA | GCCAGTTCCTCCAGATATCCA |
| *Igf1* | GAGCTGGTGGATGCTCTTCA | CTCCGAATGCTGGAGCCATA |
| *Igf1r* | ATGGAGCCTGAGAACATGGA | CCTTGTGTCCTGAGTGTCTT |
| *Il1b* | TGGCAACTGTTCCTGAACTCA | GGGTCCGTCAACTTCAAAGAAC |
| *Il6* | CCAGAAACCGCTATGAAGTTCC | GTTGTCACCAGCATCAGTCC |
| *Il8* | GGCTACTGTTGGCCCAATTAC | GCTTCATTGCCGGTGGAAA |
| *Itgav* | AAAGGCAGATGGCAAGGGAA | GGCTCCCTTCTGCTTGAGTTTA |
| *Itgb3* | CCCACCACAGGCAATCAAAA | GCGTCAGCACGTGTTTGTA |
| *Jag1* | TCCCAAGCATGGGTCTTGTA | GATGCACTTGTCGCAGTACA |
| *Lect1* | CCTGCCGATTTTCTGGCTTA | AGAGGGAGCACTGTTTCTCA |
| *Lep* | AGACCATTGTCACCAGGATCA | ATGAAGTCCAAGCCAGTGAC |
| *Mdk* | TTGCCCTCTTGGTGGTCAC | CCAGGTCCACTCCGAACAC |
| *Mmp14* | CAAGGCTGATTTGGCAACCA | GCCTTGATCTCAGTCCCAAAC |
| *Mmp2* | CGAGGACTATGACCGGGATA | GGGCACCTTCTGAATTTCCA |
| *Mmp9* | TCCCCAAAGACCTGAAAACC | GGGTGTAACCATAGCGGTAC |
| *Neat1* | GGGAAGCTGATTGCCAAGAA | ATGGTTTCAGAGCCCACAAC |
| *P54nrb* | TGGTACTCCAGCTCCTCCA | CAGCTTGGCCAAAACGTTCA |
| *Nos3* | GGGATTCTGGCAAGACAGACTA | GCAGCCAAACACCAAAGTCA |
| *Notch4* | ACCTGCTTGCAACCTTCCA | GGTGCACTCATTGACCTCCA |
| *Nrp1* | CCTGTATCCTGGGAAACTGGTA | GCCCAACATTCCAGAGCAA |
| *Nrp2* | GTGGATCAGCAGCGCTAAC | GCCATCACTCTGCAGTTTCAA |
| *PAI1 (serpinE1)* | CAGACAATGGAAGGGCAACA | GAGGTCCACTTCAGTCTCCA |
| *Pdgfa* | TGTAACACCAGCAGCGTCAA | GGCTTCTTCCTGACATACTCCA |
| *Pecam1* | GCACAGTGATGCTGAACAAC | GTCACCTTGGGCTTGGATAC |
| *Pf4* | CCAGCCTGGAGGTGATCAA | GGCAAATTTTCCTCCCATTCTTCA |
| *Pgf* | CCAATCGGGATCCACATTTCTA | GCCTTTGTCGTCTCCAGAATA |
| *Plau (upa)* | TAGCCTAGGCCTGGGGAAA | AGGCCAATCTGCACATAGCA |
| *Plg* | TGGAATTGCCCACAGTTTCC | CCGATAGTCTTTGCCATTCCC |
| *Prok2* | GGCTTGGCGTGTTTAAGGAC | GGGTCGCATTTCAAGTTCCTAC |
| *Prox1* | GCCCTCAACATGCACTACAAC | CGTGATCTGCGCAACTTCC |
| *Psf/Sfpq* | TGAAAAGCTGGCCCAGAAGAA | TGTGCCATGCTGAGCAAAAC |
| *Pspc1* | TCCCCGTGGAGCAATAAACA | ATACCCATCATTGGAGGAGGAC |
| *Ptgs1* | TATCACCTGCGGCTCTTCAA | GTTCCACGGAAGGTGGGTA |
| *S1pr1* | CGGTGTAGACCCAGAGTCC | GAGAGGCCTCCGAGAAACA |
| *SerpinF1* | AGAACCTCAAGAGTGCTTCCA | TTCTCCAGAGGGGCAACAAA |
| *Sphk1* | GGCAGCTTCTGTGAACCACTA | CAGCAGGTTCATGGGTGACA |
| *Tek* | GTTGGATGGCAATCGAATCAC | CCAGAGCAATACACCATAGGAC |
| *Tgfa* | CCCTGGCTGTCCTCATTATCA | CAGTGTTTGCGGAGCTGAC |
| *Tgfb1* | GCTGCGCTTGCAGAGATTAA | GTAACGCCAGGAATTGTTGCTA |
| *Tgfb2* | GCCCATATCTATGGAGTTCAGACA | AGCGGAAGCTTCGGGATTTA |
| *Tgfbr1* | AATTGCTCGACGCTGTTCTA | ACCGATGGATCAGAAGGTACA |
| *Thbs1* | CCCCAGAAGACATTCTCAGGAA | CGTTCACCACGTTGTTGTCA |
| *Thbs2* | GACTGCACGTCATGGTGAAC | CCCAATGAGCTCCAAAAGGAAC |
| *Tie1* | CCTTTGCTCAGATCGCACTA | CTCAAACAGCGACATGTTCAC |
| *Timp1* | TCCCCAGAAATCAACGAGACC | CATTTCCCACAGCCTTGAATCC |
| *Timp2* | GAAGAGCCTGAACCACAGGTA | TCATCCGGGGAGGAGATGTA |
| *Timp3* | CCCTTTGGCACTCTGGTCTA | ACGTGGGGCATCTTACTGAA |
| *Tnf* | CAAATGGCCTCCCTCTCATCA | TGGGCTACAGGCTTGTCAC |
| *Tymp* | GGCACACTGGATAAGCTGGAA | CAGCAGCCGACTTCCTCAA |
| *Vash1* | GGCTGCCAAGTTGGGGTGTGTT | AAACCAGGGCGTGGCTCCTGTA |
| *Vegfa* | CCAGCACATAGGAGAGATGAG | CTGGCTTTGTTCTGTCTTTCTT |
| *Vegfb* | GAGATGTCCCTGGAAGAACACA | TGGCTTCACAGCACTCTCC |
| *Vegfc* | AGACGTTCTCTGCCAGCAA | AGGCATCGGCACATGTAGTTA |
| *Vegfd (figf)* | TCCATTCAGACCCCAGAAGAA | GTGTTATCCCACAGCATGTCA |
| *Vegfr2 (Kdr)* | ATTTCACCTGGCACTCTCCA | TCCCAGGAAAGGGTTTCACA |
| *Vegfr3 (Flt4)* | CTCGCTCGGGACATCTACAAA | GGGCCATCCATTTCAGAGGAA |
| 18S | CAACTAAGAACGGCCATGCA | AGCCTGCGGCTTAATTTGAC |
