# Supplementary material for: Vasohibin1, a new mouse cardiomyocyte IRES trans-acting factor that regulates translation in early hypoxia
Source: eLife. 2019 Dec 9;8:e50094. doi: 10.7554/eLife.50094 (PMC6946400; doi:10.7554/eLife.50094)
Supplement: Supplementary file 7. — HL-1 cardiomyocytes were treated with siVASH1 of siControl and submitted to 8 hr of hypoxia or maintained in normoxia (see also Figure 8). RNA was purified from polysome fractions and from cell lysate before loading. cDNA and PCR array were performed as in Figure 1. Relative quantification (RQ) of gene expression during hypoxia was calculated using the 2–ΔΔCT method with normalization to 18S rRNA and to SiControl. mRNA levels (polysomal RNA/total RNA) are shown. When the RQ value is inferior to 1, the fold change is expressed as −1/RQ. ‘ND’ means that RNA was not detected. [file elife-50094-supp7.docx]

| **Effect of SiVASH1** | | | | |
| --- | --- | --- | --- | --- |
| **RQ=2^-ΔΔCT^** | **Polysomal/total RNA** | | | |
| **Gene name** | **Mean** | SD | **Mean** | SD |
| ***Akt1*** | **-1.08** | 0.10 | **1.70** | 0.38 |
| ***Ang*** | **1.77** | 0.47 | **-3.15** | 0.10 |
| ***Angpt1*** | **-1.27** | 0.10 | **8.31** | 1.98 |
| ***Angptl4*** | **-3.02** | 0.01 | **ND** |  |
| ***Anpep*** | **1.10** | 0.20 | **ND** |  |
| ***Apelin*** | **-12.53** | 0.01 | **ND** |  |
| ***Atp2a2*** | **-1.30** | 0.15 | **3.90** | 0.10 |
| ***Ccl21a*** | **ND** |  | **9661.44** | 2449 |
| ***Col18a1*** | **-2.05** | 0.11 | **1.45** | 0.15 |
| ***Col4a3*** | **1.70** | 0.29 | **-22.32** | 0.01 |
| ***Ctgf*** | **-2.66** | 0.05 | **7.83** | 0.03 |
| ***Cxcl1*** | **-1.61** | 0.25 | **-5.95** | 0.04 |
| ***Cxcl10 (Inp10)*** | **-16.31** | 0.02 | **ND** |  |
| ***Cyr 61*** | **-1.11** | 0.14 | **1.26** | 0.46 |
| ***Edn1*** | **2.54** | 0.19 | **1.18** | 0.14 |
| ***Efna1*** | **-1.42** | 0.14 | **-1.43** | 0.07 |
| ***Efnb2*** | **-1.41** | 0.03 | **-3.42** | 0.05 |
| ***Egf*** | **-2.68** | 0.12 | **ND** |  |
| ***Eng*** | **-1.36** | 0.01 | **ND** |  |
| ***Ephb4*** | **-1.33** | 0.00 | **-4.00** | 0.06 |
| ***Erbb2(Her2)*** | **-1.39** | 0.11 | **-2.49** | 0.08 |
| ***F3*** | **2.09** | 0.44 | **1.22** | 0.16 |
| ***Fgf1*** | **-1.99** | 0.11 | **ND** |  |
| ***Fgfr3*** | **-1.89** | 0.05 | **-1.37** | 0.09 |
| ***Fibrillarin*** | **-1.60** | 0.10 | **-1.90** | 0.08 |
| ***Fn1*** | **-2.46** | 0.10 | **4.02** | 0.68 |
| ***Hif1a*** | **1.38** | 0.16 | **4.82** | 1.13 |
| ***Hif2a*** | **-2.21** | 0.03 | **ND** |  |
| ***Hnrnpm*** | **1.01** | 0.17 | **3.91** | 0.67 |
| ***Hpse*** | **2.20** | 0.02 | **-1.08** | 0.03 |
| ***Igf1*** | **2.71** | 1.08 | **1.38** | 0.29 |
| ***Igf1r*** | **-1.47** | 0.04 | **-3.14** | 0.06 |
| ***Itgav*** | **-1.17** | 0.13 | **ND** |  |
| ***Itgb3*** | **1.96** | 0.20 | **-1.66** | 0.00 |
| ***Kdr*** | **-1.35** | 0.15 | **15.02** | 3.72 |
| ***Mmp14*** | **1.38** | 0.02 | **-2.84** | 0.12 |
| ***Neat-1*** | **-2.86** | 0.05 | **34.05** | 2.09 |
| ***Nos3*** | **-4.54** | 0.08 | **ND** |  |
| ***Nrp1*** | **1.21** | 0.09 | **1.19** | 0.30 |
| ***Nrp2*** | **-1.28** | 0.14 | **-4.68** | 0.02 |
| ***P54nrb*** | **-1.38** | 0.01 | **-1.21** | 0.23 |
| ***Pdgfa*** | **-1.11** | 0.10 | **2.91** | 0.35 |
| ***Pecam1*** | **-2.53** | 0.01 | **ND** |  |
| ***Pf4*** | **-3.75** | 0.03 | **ND** |  |
| ***Pgf*** | **-1.70** | 0.04 | **ND** |  |
| ***Plau(Upa)*** | **-1.53** | 0.05 | **ND** |  |
| ***Prok2*** | **ND** |  | **3.38** | 2.45 |
| ***Prox1*** | **2.24** | 0.52 | **7.26** | 1.33 |
| ***Psf/Sfpq*** | **1.85** | 0.20 | **2.46** | 0.24 |
| ***Pspc1*** | **-2.27** | 0.08 | **3.17** | 0.54 |
| ***Rnasep*** | **-1.66** | 0.12 | **21.24** | 1.70 |
| ***Rpl11*** | **-3.23** | 0.10 | **-1.31** | 0.14 |
| ***Serpinf1*** | **-2.32** | 0.15 | **ND** |  |
| ***Sphk1*** | **-3.85** | 0.04 | **ND** |  |
| ***Tek*** | **12.03** | 3.59 | **25.35** | 3.48 |
| ***Tgfb1*** | **-2.27** | 0.03 | **11.88** | 3.07 |
| ***Tgfb2*** | **4.51** | 0.55 | **-1.01** | 0.19 |
| ***Tgfbr1*** | **-2.21** | 0.17 | **1.24** | 0.25 |
| ***Thbs1*** | **7.48** | 2.32 | **2.37** | 0.29 |
| ***Thbs2*** | **-1.38** | 0.08 | **-3.70** | 0.07 |
| ***Timp1*** | **1.24** | 0.34 | **-3.70** | 0.06 |
| ***Timp2*** | **1.91** | 0.10 | **-2.78** | 0.10 |
| ***Timp3*** | **-1.70** | 0.17 | **ND** |  |
| ***Vegfa*** | **1.41** | 0.19 | **10.07** | 2.93 |
| ***Vegfb*** | **1.40** | 0.21 | **5.63** | 0.95 |
| ***Vegfd (Figf)*** | **2.90** | 0.39 | **ND** |  |
