# Supplementary material for: Vasohibin1, a new mouse cardiomyocyte IRES trans-acting factor that regulates translation in early hypoxia
Source: eLife. 2019 Dec 9;8:e50094. doi: 10.7554/eLife.50094 (PMC6946400; doi:10.7554/eLife.50094)
Supplement: Supplementary file 8. — (A) Sequence of the hairpin inserted in the bicistronic lentivector between the LucR and Luc+ genes. The LucR stop codon and the Luc+ start codon are indicated. The complementary sequences are indicated in red and in blue, respectively. (B) Sequences of the four siRNAs present in the siControl and siVASH1 smartpools. [file elife-50094-supp8.docx]

Hantelys et al, Supplementary File 8

A/

taaACTAGACGCGCTCTCCGTGAACTAGCGTAGCTGACCGATATCGGTCAGCTACGCTAGTTCACGGAGAGCGCGACTAGTGGATCCatg

B/

| siControl (5’ -> 3’) | siVASH1 (5’->3’) |
| --- | --- |
| ACCAAAUGUACAGCUGAUU | GACACUAGGACCCUUAAAU |
| ACCAAAUGUACAAAAGACU | CGAAGUUCUGGAUAAAGAG |
| ACCAAAUGUACAAAAGGAU | CCCUCCUGGACUACAUGUU |
| ACCAAAUGUACAACACACU | CAUGUUAGUGUGUCCCUGU |
