# Supplementary material for: Vasohibin1, a new mouse cardiomyocyte IRES trans-acting factor that regulates translation in early hypoxia
Source: eLife. 2019 Dec 9;8:e50094. doi: 10.7554/eLife.50094 (PMC6946400; doi:10.7554/eLife.50094)
Supplement: Transparent reporting form [file elife-50094-transrepform.docx]

***eLife’s* transparent reporting form**

We encourage authors to provide detailed information *within their submission* to facilitate the interpretation and replication of experiments. Authors can upload supporting documentation to indicate the use of appropriate reporting guidelines for health-related research (see [EQUATOR Network](http://www.equator-network.org/%20)), life science research (see the [BioSharing Information Resource](https://biosharing.org/)), or the [ARRIVE guidelines](http://www.plosbiology.org/article/info:doi/10.1371/journal.pbio.1000412) for reporting work involving animal research. Where applicable, authors should refer to any relevant reporting standards documents in this form.

If you have any questions, please consult our Journal Policies and/or contact us: [editorial@elifesciences.org](mailto:editorial@elifesciences.org).

**Sample-size estimation**

- You should state whether an appropriate sample size was computed when the study was being designed
- You should state the statistical method of sample size computation and any required assumptions
- If no explicit power analysis was used, you should describe how you decided what sample (replicate) size (number) to use

Please outline where this information can be found within the submission (e.g., sections or figure legends), or explain why this information doesn’t apply to your submission:

The appropriate sample size was decided according to the feasibility of each experiment, in order to be able to make statistics. Except for one fluidigm experiments (PCR array), all data were obtained from nine biological replicates. Each biological replicate value resulted from three technical replicates. Details are indicated in figure and table legends.

**Replicates**

- You should report how often each experiment was performed
- You should include a definition of biological versus technical replication
- The data obtained should be provided and sufficient information should be provided to indicate the number of independent biological and/or technical replicates
- If you encountered any outliers, you should describe how these were handled
- Criteria for exclusion/inclusion of data should be clearly stated
- High-throughput sequence data should be uploaded before submission, with a private link for reviewers provided (these are available from both GEO and ArrayExpress)

Please outline where this information can be found within the submission (e.g., sections or figure legends), or explain why this information doesn’t apply to your submission:

-> Definition: Biological replicates correspond to cell culture flasks or wells that were treated separately. Technical replicates correspond to different technical measurement (luminescence activity, PCR reaction …) made on a biological replicate.

-> Fluidigm PCR array, transcriptome :(Fig. 1, Fig. 1-sup 1, Fig. 3-sup 1, Fig. 8, , Suppl. File 1) : RT qPCR measurements were done from one experiment, with 7 points of kinetics, with biological triplicates for each gene and technical triplicates for each biological replicate.

-> Fluidigm array, polysomes (Fig. 2, Fig. 3B, Suppl. File 2, Fig. 8, Suppl. File 7) : for Suppl File 2 and corresponding figures, two independent experiments RQ1 (4h of hypoxia) and RQ2 (4h and 24h) were performed. RQ1 was performed with 3 biological replicates and 3 technical replicates for each biological replicate. RQ2 with 2 biological replicates and 2 technical replicates for each biological replicate. For Suppl File 7 and Fig. 8 only one array with siVASH1 and siControl was performed in normoxia and at 8 hours of hypoxia. The RQ was measured as for Suppl File 2.

-> Luminescence measurements (Figure 4, Figure 7 C-D , Suppl File 3, Suppl File 5): 9 biological replicates. N= 9. All raw values are indicated in Suppl File 3 and 5. Each raw value corresponds to the mean of three technical replicates (luciferase measurement made 3 times on the same sample).

-> Surface plasmonic resonance and mass spectrometry (Figure 5, Suppl File 4). The list of peptides results from one experiment of BIAMS, but with 2 times of hypoxia (each of them corresponding to 6 pooled injections in the Biacore apparatus. One experiment for KD measurement of recombinant VASH1 binding to RNA, but for three IRESs.

-> VASH1 expression and localization (Figure 6 and 7A). For VASH1 expression (total RNA and polysomes): values from RQ2 PCR array (Suppl File 2), 2 biological replicates with two technical replicates each. For VASH1 protein localization : three independent experiments. Focus quantification was made on 4-5 images with a total cell number of 149 in normoxia and 178 in hypoxia.

-> Protein expression by capillary Western (Fig. 2B, 4C, 6C, 7B and Fig. 2-sup 1): experiments were reproduced 2 or 3 times depending on the antibody from independent cell extracts.

-> VASH1 half-life measurement (Fig. 7-sup 1): Three independent experiments.

Details of replicates are indicated in figure and table legends.

No data were excluded: the only data excluded were when cells became insensitive to hypoxia after too many passages and data were not exploitable. No values were excluded in the experiments to make statistics.

Importantly, the main data of the paper, in Figure 4 and 7, were reproduced by three independent experimenters.

Lentivector plasmid sequences are available on Dryad.

**Statistical reporting**

- Statistical analysis methods should be described and justified
- Raw data should be presented in figures whenever informative to do so (typically when N per group is less than 10)
- For each experiment, you should identify the statistical tests used, exact values of N, definitions of center, methods of multiple test correction, and dispersion and precision measures (e.g., mean, median, SD, SEM, confidence intervals; and, for the major substantive results, a measure of effect size (e.g., Pearson's r, Cohen's d)
- Report exact p-values wherever possible alongside the summary statistics and 95% confidence intervals. These should be reported for all key questions and not only when the p-value is less than 0.05.

Please outline where this information can be found within the submission (e.g., sections or figure legends), or explain why this information doesn’t apply to your submission:

All statistical analyses were performed using one-way Anova with Tukey’s comparisons test (Fig. 6) or two-tailed Student's t-test (Fig. 3) or Mann-Whitney test (Fig. 4 and 7) and are expressed as mean +- standard deviation, *p<0.05, **p<0.01, ***<0.001, ****<0.0001. This indicated in each figure legend.

All SD and p-values are reported for luminescence activity measurements in Suppl File 3 and 5.

(For large datasets, or papers with a very large number of statistical tests, you may upload a single table file with tests, Ns, etc., with reference to sections in the manuscript.)

**Group allocation**

- Indicate how samples were allocated into experimental groups (in the case of clinical studies, please specify allocation to treatment method); if randomization was used, please also state if restricted randomization was applied
- Indicate if masking was used during group allocation, data collection and/or data analysis

Please outline where this information can be found within the submission (e.g., sections or figure legends), or explain why this information doesn’t apply to your submission:

Not applicable

**Additional data files (“source data”)**

- We encourage you to upload relevant additional data files, such as numerical data that are represented as a graph in a figure, or as a summary table
- Where provided, these should be in the most useful format, and they can be uploaded as “Source data” files linked to a main figure or table
- Include model definition files including the full list of parameters used
- Include code used for data analysis (e.g., R, MatLab)
- Avoid stating that data files are “available upon request”

Please indicate the figures or tables for which source data files have been provided:

Source data are provided in Supplementary Files for Graphs of Fig. 1, 2, 3, 4, 5, 7 and 8.
